# Improving the Predictions of Computational Models of Convection-Enhanced Drug Delivery by Accounting for Diffusion Non-gaussianity

**DOI:** 10.3389/fneur.2018.01092

**Published:** 2018-12-18

**Authors:** Eirini Messaritaki, Suryanarayana Umesh Rudrapatna, Greg D. Parker, William P. Gray, Derek K. Jones

**Affiliations:** ^1^BRAIN Biomedical Research Unit, Cardiff University, Cardiff, United Kingdom; ^2^Cardiff University Brain Research Imaging Centre (CUBRIC), School of Psychology, Cardiff, United Kingdom; ^3^School of Psychology, Cardiff University, Cardiff, United Kingdom; ^4^Experimental MRI Centre (EMRIC), School of Biosciences, Cardiff University, Cardiff, United Kingdom; ^5^School of Medicine, Cardiff University, Cardiff, United Kingdom; ^6^Neuroscience and Mental Health Research Institute, Cardiff University, Cardiff, United Kingdom; ^7^Faculty of Health Sciences, School of Psychology, Australian Catholic University, Melbourne, VIC, Australia

**Keywords:** convection-enhanced drug delivery, diffusion MRI, non-Gaussian diffusion, neurodegenerative diseases, computational fluid dynamics, Parkinson's disease, Huntington's disease, epilepsy

## Abstract

Convection-enhanced delivery (CED) is an innovative method of drug delivery to the human brain, that bypasses the blood-brain barrier by injecting the drug directly into the brain. CED aims to target pathological tissue for central nervous system conditions such as Parkinson's and Huntington's disease, epilepsy, brain tumors, and ischemic stroke. Computational fluid dynamics models have been constructed to predict the drug distribution in CED, allowing clinicians advance planning of the procedure. These models require patient-specific information about the microstructure of the brain tissue, which can be collected non-invasively using magnetic resonance imaging (MRI) pre-infusion. Existing models employ the diffusion tensor, which represents Gaussian diffusion in brain tissue, to provide predictions for the drug concentration. However, those predictions are not always in agreement with experimental observations. In this work we present a novel computational fluid dynamics model for CED that does not use the diffusion tensor, but rather the diffusion probability that is experimentally measured through diffusion MRI, at an individual-participant level. Our model takes into account effects of the brain microstructure on the motion of drug molecules not taken into account in previous approaches, namely the restriction and hindrance that those molecules experience when moving in the brain tissue, and can improve the drug concentration predictions. The duration of the associated MRI protocol is 19 min, and therefore feasible for clinical populations. We first prove theoretically that the two models predict different drug distributions. Then, using *in vivo* high-resolution diffusion MRI data from a healthy participant, we derive and compare predictions using both models, in order to identify the impact of including the effects of restriction and hindrance. Including those effects results in different drug distributions, and the observed differences exhibit statistically significant correlations with measures of diffusion non-Gaussianity in brain tissue. The differences are more pronounced for infusion in white-matter areas of the brain. Using experimental results from the literature along with our simulation results, we show that the inclusion of the effects of diffusion non-Gaussianity in models of CED is necessary, if reliable predictions that can be used in the clinic are to be generated by CED models.

## 1. Introduction

Convection-enhanced delivery (CED) is an innovative method of drug delivery to the human brain that aims to increase the efficiency of drug treatment for many disorders of the central nervous system. The innovation of the technique lies in the fact that, instead of being delivered through the bloodstream, the drug is infused directly into the brain tissue via catheters implanted in the brain, while a constant pressure is applied at the tip of the catheter. CED is very invasive and requires constant monitoring of the patient during the infusion. At the same time, it allows clinicians to bypass the blood-brain barrier that can inhibit the reach of large-macromolecule drugs to the pathology, it results in the drug reaching an extended volume within a few hours of infusion, and it limits the drug concentration in healthy tissue, thus limiting the drug-related side effects that the patient can suffer (1, 2). CED was studied experimentally in animals as early as the 1990s (3, 4) and later (5), and a theoretical model for it was first proposed in 1994 (6). More recently, CED has been used on patients that suffer from brain tumors (7–10), epilepsy (1), and Parkinson's disease (PD) (2). The method also presents potential for use in Huntington's disease (HD), Alzheimer's disease (AD) (2), and ischemic stroke (11). Studies that aim at monitoring the distribution of the drug in real time through magnetic resonance imaging (MRI) have also taken place (9, 12). A thorough review of the scope, technology and proposed developments of CED was presented by Raghavan et al. (13).

In order for CED to be effective, clinicians need to know the initial catheter setup (the catheter location and the velocity and pressure of infusion of the drug) that will allow the drug to cover the entirety of the pathology in question while minimizing dose to healthy brain tissue. To that end, computational fluid dynamics (CFD) models that predict the drug's distribution in the brain for a given initial catheter setup have been developed. CFD models that rely on different metrics for brain tissue characterization or which assume different mechanisms for the movement of drug molecules would predict different drug distributions for the same initial catheter setup. Inversely, to achieve a desired drug distribution, different CFD models would advocate different initial catheter setups. Accurate models are therefore needed to correctly inform the catheter setup to be used in the clinical setting. Computational models have been developed by various groups to predict the drug distribution resulting from CED in rabbit brain (14, 15), in pig brain (5), in rat spinal cord (16–18), in tumor animal models (19, 20), and in the human brain (21, 22). In all these studies it is important to account for all details of the tissue microstructure that affect the motion of the drug molecules, in order to provide accurate predictions that eliminate the uncertainty in targeting the structures of interest.

Modeling CED is inherently challenging because the drug moves in the anisotropic and heterogeneous brain tissue, and that anisotropy and heterogeneity needs to be accurately reflected in the CFD models through appropriate microstructural representations. MRI and diffusion-MRI (dMRI) allow non-invasive imaging of the human brain. The CFD models in the previously mentioned studies use the diffusion tensor (DT) (23), measured through dMRI, to map the brain microstructure. However, even though the DT is a powerful tool for studying microstructure in the human brain (24, 25), it only encompasses Gaussian diffusion for the water molecules (26–30). This limitation needs to be considered carefully when modeling CED, because the non-Gaussian character of diffusion that is evidenced in the motion of water molecules in the brain, and which includes phenomena observed *in vivo* such as restriction and hindrance, will also affect the drug molecules.

In this work, we use a theoretical fluid dynamics model that was recently presented in the literature (31, 32) to develop a CFD model that overcomes the limitations of the DT approach and improves the predictions of the concentration of drugs infused through CED into brain tissue by accounting for phenomena not included in the DT representation. In contrast to CFD models that use the DT, our new framework uses the diffusion displacement covariance tensor of water molecules in the brain to predict how the drug molecules will move in the brain tissue. This captures the second-order effects of restricted and hindered diffusion that molecules can experience in brain tissue, that are not represented by the DT (30). Importantly, we prove theoretically that the predictions given by our more comprehensive model differ significantly from those given by the model that uses the DT. We then use human high-resolution diffusion MRI data to perform CFD simulations of drug delivery using both models and quantify those differences. In section 2, we present our new theoretical framework for constructing the new CED fluid dynamics model, and detail the differences between this framework and that reported previously in the literature. We also describe the details of the MR protocol, the data processing and the calculation of the microstructural parameters needed in our CFD model. In section 3 we compare the microstructural measures used by the two different CFD models, present the results that the models give for the drug concentration in the human brain for different infusion sites and compare those results. We also present evidence from existing literature that accounting for diffusion non-Gaussianity can improve the predictions of CED models. In section 4 we discuss the results and give some directions for future work. The detailed theoretical derivation of the CFD framework is presented in Appendix A.

## 2. Materials and Methods

### 2.1. Fluid Dynamics Models

In this section we describe the equations of the fluid dynamics model that is the basis of the novel CFD model we use.

#### 2.1.1. Proposed Formalism

Berkowitz et al. (31) presented a framework that describes the movement of fluid molecules under the influence of diffusion and convection, consisting of the equations:

(1) $$\begin{array}{l} \nabla \cdot \text{v}\left(\text{r}\right)=0 \end{array}$$

(2) $$\begin{array}{l} \text{v}\left(\text{r}\right)=-\text{T}\left(\text{r}\right)\nabla p\left(\text{r}\right) \end{array}$$

(3) $$\begin{array}{l} \varphi (r)\frac{\partial C\left(r,t\right)}{\partial t}=-\nabla \cdot \left[v(r)C(r,t)\right]+\nabla \cdot \;\left[\varphi \left(r\right)R\left(r\right)\;\cdot \;\nabla C\left(r,t\right)\right] \end{array}$$

where **v**(**r**) is the drug velocity at location **r**, *p*(**r**) is the pressure at **r**, and *C*(**r**, *t*) is the drug concentration at **r** at time *t*. The tensor **R**(**r**) is given by (31):

(4) $$\begin{array}{l} \text{R}\left(\text{r}\right)=\frac{1}{2}\underset{{\text{r}}^{\prime }}{\sum }w_{\text{d}}\left(\text{r},{\text{r}}^{\prime }\right)\left({\text{r}}^{\prime }-\text{r}\right)\left({\text{r}}^{\prime }-\text{r}\right) \end{array}$$

where $w_{\text{d}}\left(\text{r},{\text{r}}^{\prime }\right)$ is the transition rate of a fluid molecule from position **r** to position **r**′ in a given time *due to diffusion only*. The tensor **T**(**r**) is an effective permeability tensor and is related to the tensor **R**(**r**) by the equation (31):

(5) $$\begin{array}{l} \text{T}\left(\text{r}\right)\equiv \frac{\text{R}\left(\text{r}\right)}{\hat{\lambda }}. \end{array}$$

In this equation, $\hat{\lambda }$ is a factor that gives the relative scale of the diffusion vs. the convection process, for the porous medium in which the fluid is moving. These equations are derived by considering the transition of fluid molecules between neighboring points of the porous medium due to diffusion and convection, and then Taylor-expanding the transition rate and the pressure difference between those points to second order in their distance. The first term on the right-had side of Equation (3) is the convection contribution, while the second term on the right-hand side is the diffusion contribution. The detailed derivation of these equations is given in Appendix A of this paper as it was originally presented by Berkowitz et al. (31).

#### 2.1.2. Diffusion-Tensor-Based Formalism

The equations that have been previously used in the literature ((16, 17), etc.) to describe the motion of a fluid in brain tissue due to diffusion and convection are:

(6) $$\begin{array}{l} \nabla \cdot \text{v}\left(\text{r}\right)=0 \end{array}$$

(7) $$\begin{array}{l} \text{v}\left(\text{r}\right)=-\text{K}\left(\text{r}\right)\nabla p\left(\text{r}\right) \end{array}$$

(8) $$\begin{array}{l} \varphi (r)\frac{\partial C\left(r,t\right)}{\partial t}=-\nabla \cdot \left[v(r)C(r,t)\right]+\nabla \cdot \left[\varphi \left(r\right)D\left(r\right)\;\cdot \;\nabla C\left(r,t\right)\right], \end{array}$$

Equation (6) is the continuity equation, and Equation (7) is Darcy's law that relates the fluid velocity to the pressure gradient. Equation (8) is the fluid transport equation, in which the first term of the right-hand side is the convection contribution to the rate of change of the concentration, while the second term is the diffusion contribution. **D**(**r**) is the drug diffusion tensor (DT), derived by appropriately calibrating the water DT (22, 33). The water DT, **D**_w_(**r**), can be measured through dMRI (23) and provides a measure of how far and in what direction the water molecules in brain tissue will move due to diffusion, under the assumption of Gaussian diffusion. It can be calculated through the equation

(9) $$\begin{array}{l} S_{\text{k}}=S_{0}e^{-b{\text{g}}_{\text{k}}^{\text{T}}{\text{D}}_{\text{w}}{\text{g}}_{\text{k}}} \end{array}$$

where *S*_0_ is the MR signal with no diffusion weighting, *S*_**k**_ is the signal when a diffusion weighting gradient **g**_**k**_ has been applied, and *b* is the strength of the diffusion weighting [for more details on dMRI we refer the reader to the plethora of relevant papers, for example (23, 34–36) and references therein.] **K**(**r**) is the hydraulic conductivity tensor of the drug, which can also be derived by appropriate calibration of the water DT (22, 33), and φ(**r**) is the porosity of the tissue.

In the following, we refer to the model that employs the DT as the D-model and the model that employs the tensor **R** as the R-model.

#### 2.1.3. Differences Resulting From Considering the Effects of Non-Gaussianity

The transport Equations (3) and (8) indicate that there are differences between the concentration predicted when accounting for diffusion non-Gaussianity to that predicted by the simpler model that only encompasses Gaussian diffusion. By rewriting those equations using the subscripts “R” and “D” to indicate the model the quantities refer to, and skipping the explicit time and spatial dependence for notational clarity, we get:

(10) $$\begin{array}{l} \varphi \frac{\partial C_{\text{R}}}{\partial t}=-\nabla \;\cdot \;\left(v_{\text{R}}C_{\text{R}}\right)+\;\nabla \;\cdot \;\left(\varphi R\cdot \nabla C_{\text{R}}\right) \end{array}$$

and

(11) $$\begin{array}{l} \varphi \frac{\partial C_{\text{D}}}{\partial t}=-\nabla \;\cdot \;\left(v_{\text{D}}C_{\text{D}}\right)+\nabla \;\cdot \;\left(\varphi D\;\cdot \;\nabla C_{\text{D}}\right). \end{array}$$

Subtracting the two equations and setting Δ*C* = *C*_R_ − *C*_D_ and Δ**v** = **v**_R_ − **v**_D_, we get

(12) $$\begin{array}{l} \varphi \frac{\partial \left(\Delta C\right)}{\partial t}+\nabla \;\cdot \;\left(v_{\text{R}}\Delta C\right)-\nabla \;\left[\varphi R\;\cdot \;\nabla \left(\Delta C\right)\right]\\ =-\nabla \;\cdot \;\left(\Delta v\;\cdot \;C_{\text{D}}\right)\;+\;\nabla \;\cdot \;\left[\varphi \left(R-D\right)\cdot \nabla C_{\text{D}}\right]. \end{array}$$

Equation (12) is a time evolution equation for the difference in concentrations. Both terms on the right-hand side of the equation are proportional to the divergence of the difference **R** − **D**. This equation conveys the fact that the concentration predicted by the R-model differs from that predicted by the D-model, and the difference depends on several parameters, such as the duration of infusion, the location under consideration, and how far and in what direction that location is in relation to the infusion site. The equation also conveys the complexity of the dependence of the difference in concentration in those parameters. For example, it is clear that the difference between the tensors **R** and **D** will impact Δ*C*. Specifically, for any given location of interest in the brain, it will be the cumulative difference of those tensors from the infusion point to the location of interest for all the paths that the fluid molecules follow between those two points that will impact Δ*C*. Also, the fact that Δ*C* evolves with time implies that any given brain location will be impacted differently at different times during the infusion process. While the main aim of our work is to provide a CFD model that gives predictions that are more accurate than existing ones by accounting for all the phenomena that affect the motion of the drug molecules, quantifying the differences predicted by Equation (12) and understanding the implications they have on the clinical practice of CED is the additional scope of this manuscript.

From a physics perspective, the difference between the two formalisms lies in the assumptions made about the diffusion and convection processes in brain tissue. In the former framework, the DT represents Gaussian diffusion. However, as explained earlier, diffusion in brain tissue is predominantly not Gaussian (26–30). By employing the tensor **R** we incorporate the second-order effects of the restricted and hindered diffusion in this analysis. These effects are evident in the diffusion of water molecules, as will become clear by comparing the tensors **D** and **R** in section 3.1, and will also affect the drug molecules. Importantly, these differences affect not only the diffusion process during CED, but also the convection process, since the tensors **K** and **T** that relate the velocity to the pressure difference are proportional to the tensors **D** and **R**, respectively. In other words, by better modeling the restriction and hindrance that the molecules would encounter in their diffusive motion, we are also improving the modeling of the convective motion of the molecules.

### 2.2. Calculation of the Tensors

The new framework presented in this work requires calculation of the tensor **R**(**r**) for the diffusing motion of the drug molecules in the anisotropic brain tissue. The sum in Equation (4) is over all possible **r**′. Converting the sum to an integral we have:

(13) $$R(r)=\frac{1}{2}\frac{1}{\text{ dV}}\int_{{\mathbb{R}}^{3}}w_{\text{d}}(r,r^{\prime })(r^{\prime }-r)(r^{\prime }-r)\text{ d}r^{\prime }.$$

The rate w_d_(**r**, **r**′) that is required for the computation of the tensor **R**(**r**) is equal to the proportion of particles that start at position **r** and reach **r**′ exclusively due to the diffusion process, in the diffusion time Δ, divided by the time Δ. A quantity that is related to this rate and is measurable through dMRI is the diffusion propagator P(**r**|**r**′, Δ) (34, 37, 38).

If we assume that at time 0 there are *N* particles at **r** and that a time Δ later $N_{\text{r},{\text{r}}^{\prime }}$ of those particles reach **r**′, then the propagator (probability that a molecule at **r** will reach **r**′ in the diffusion time Δ) is

(14) $$\begin{array}{l} \text{P}\left(\text{r}|{\text{r}}^{\prime },\Delta \right)=\frac{N_{\text{r},{\text{r}}^{\prime }}}{N}\frac{1}{\text{dV}}. \end{array}$$

The diffusion-induced transition rate at which particles arrive at **r**′ from **r** is equal to the proportion of particles that arrive at **r**′ from **r** in the diffusion time Δ. Therefore,

(15) $$\begin{array}{l} w_{\text{d}}\left(\text{r},{\text{r}}^{\prime }\right)=\frac{N_{\text{r},{\text{r}}^{\prime }}}{N}\frac{1}{\Delta }=\text{P}\left(\text{r}|{\text{r}}^{\prime },\Delta \right)\frac{\text{d}\text{V}}{\Delta }. \end{array}$$

Using this expression for the transition rate in Equation (13) we get:

(16) $$\begin{array}{l} \text{R}\left(\text{r}\right)=\frac{1}{2\Delta }\int_{{\mathbb{R}}^{3}}\text{P}\left(\text{r}|{\text{r}}^{\prime },\Delta \right)\left({\text{r}}^{\prime }-\text{r}\right)\left({\text{r}}^{\prime }-\text{r}\right)\text{d}{\text{r}}^{\prime }. \end{array}$$

The tensor **R**, therefore, captures the covariance of the diffusion displacement. This tensor's eigenvectors determine the principal diffusion directions in the porous medium and its diagonal elements are the mean-squared displacements along the *x*, *y*, and *z* directions, respectively. As such, the formalism captures all forms of diffusive displacements, not just those captured by the DT formalism, and that is a direct consequence of the fact that the probability that the molecules will move along a given direction is experimentally measured.

Following Tuch et al. (33), we can calculate this tensor for the drug of interest by appropriate calibration of the equivalent tensor for water, as will be described in section 2.3.2. The covariance matrix of the diffusion displacement of the water has been proposed as a useful measure for characterizing brain microstructure (39, 40). Here we use the method proposed by Ning et al. (39) for calculating **R**, which uses Gaussian radial basis functions. For a detailed description of the methodology, we refer the reader to that paper (39). We will present details on the characteristics of the tensor **R** in section 3.1.

The calculation of the tensor **T** is straightforward once **R** has been calculated and entails a calibration of the tensor **R** in the same way that **K** is calibrated from **D** (22).

### 2.3. Scanning, Data Processing, and Microstructural Metrics

#### 2.3.1. MR Scanning and Preprocessing

A healthy participant (age range 26–30 years) was scanned in order to obtain the MR data used in our analysis. The participant gave informed written consent and all procedures were approved by the local ethics committee.

The MRI data were acquired at the Cardiff University Brain Research Imaging Centre (CUBRIC) on a 3T Siemens Connectom scanner with 300 mT/m gradients. The scanning session consisted of a T1-weighted scan and a high angular resolution diffusion imaging (HARDI) scan. The T1-weighted scan used the MPRAGE sequence (41) with parameters: TR = 2.3 s, TE = 2.2 ms, TI = 850 ms, field of view 256 × 256 × 192 mm^3^, and isotropic resolution of 1 mm^3^. The HARDI scan (42) used a multiband-EPI diffusion sequence on two shells, *b* = 1, 200 s/mm^2^ and *b* = 2, 400 s/mm^2^, with 61 isotropically-distributed diffusion gradient directions for each shell, TR = 6.2 s, TE = 63 ms, field of view 216 × 216 × 132 mm^3^, and isotropic resolution of (1.2mm)^3^. In the diffusion data, there were 180 coronal slices, 180 sagittal slices, and 110 axial slices. In order to make the Figures in subsequent sections clear, we note that we coronal slices increase in number from anterior to posterior, the sagittal slices from left to right and the axial slices from bottom to top. The total scanning time was 19 min.

The diffusion-weighted HARDI data were corrected for distortions induced by the diffusion-weighted gradients, gradient non-linearity, artifacts due to head motion, and due to EPI- induced geometrical distortions. The diffusion images were co-registered to the T1-weighted image, after down-sampling the latter to the diffusion-weighted data. The MR scanning parameters are listed in Table 1.

**Table 1:** MR scanning parameters.

**MR sequence**	**Parameter**	**Value**
MPRAGE	TR	2.3 s
	TE	2.2 ms
	TI	850 ms
	FOV	256 × 256 × 192 mm^3^
	Resolution	1 mm isotropic
HARDI	*b*-values	1, 200 and 2, 400 s/mm^2^
	Gradient directions per b-shell	61
	TR	6.2 s
	TE	63 ms
	FOV	216 × 216 × 132 mm^3^
	Resolution	1.2 mm isotropic

We used the FSL package (43, 44) to perform brain extraction and to identify voxels that contain predominantly gray matter (GM), white matter (WM), or cerebrospinal fluid (CSF).

#### 2.3.2. Calculation of the DT and of R

To calculate the tensors **R** and **D** for water we followed the method described by Ning et al. (39) and used the code provided by them at https://github.com/LipengNing/RBF-Propagator, appropriately adapted for our dataset. It was explained in section 2.1 that, in contrast to **D**_w_ which represents Gaussian diffusion, the tensor **R**_w_ uses the MR-measured probability of diffusion in all directions thus incorporating the second-order effects of restricted and hindered diffusion. Two qualities of the tensors were expected to affect how the drug distributions resulting from the two frameworks compare with each other: (a) the actual values of the components of the two tensors, which determine how quickly the drug spreads, with higher values resulting in faster spread of the drug, and (b) the anisotropies of the two tensors, which determine how much drug will spread along each direction. For that reason, we compared the values of the corresponding components of the two tensors, their fractional anisotropies (FA) and their mean diffusivities (MD). In order to compare the range of the diffusion process as predicted by each of the two tensors **R**_w_ and **D**_w_, we calculated the ratio of their traces Tr(**R**_w_)/Tr(**D**_w_) for each voxel, noting that the ratio provides information about the relative ranges of the diffusive motion of the water molecules in a given voxel, without conveying any information about the range in that voxel in comparison to the range in other voxels.

Both these tensors were calculated for the water molecules, and therefore a calibration procedure was needed to convert them to the tensors that describe the movement of drug molecules, as is explained in Linninger et al. (22). There is uncertainty in the specific values that these tensors assume in the human brain, as discussed in Stoverud et al. (45). In this work we followed the method described in Linninger et al. (22) and Stoverud et al. (45), which is based on the work of Avellaneda and Torquato (46), to perform the required calibration. Specifically, we first decomposed the water diffusion tensor into its eigenvectors ξ and eigenvalues λ_*i*_, *i* = 1, 2, 3, for each voxel:

(17) $$\begin{array}{l} {\text{D}}_{\text{w}}=\xi_{\text{D}}\cdot \Lambda_{\text{D}}\cdot \xi_{\text{D}}^{\text{T}} \end{array}$$

where

(18) $$\begin{array}{l} \Lambda_{\text{D}}=\left[\begin{array}{ccc} \lambda_{1}^{\text{D}} & 0 & 0\\ 0 & \lambda_{2}^{\text{D}} & 0\\ 0 & 0 & \lambda_{3}^{\text{D}} \end{array}\right]. \end{array}$$

Then we rescaled the eigenvalues by their average ${\bar{\lambda }}^{\text{D}}=\frac{1}{3}\left(\lambda_{1}^{\text{D}}+\lambda_{2}^{\text{D}}+\lambda_{3}^{\text{D}}\right)$, to get ${\bar{\lambda }}_{i}^{\text{D}}=\lambda_{i}^{\text{D}}/\bar{\lambda^{\text{D}}}$ and

(19) $$\begin{array}{l} {\bar{\Lambda }}_{\text{D}}=\left[\begin{array}{ccc} {\bar{\lambda }}_{1}^{\text{D}} & 0 & 0\\ 0 & {\bar{\lambda }}_{2}^{\text{D}} & 0\\ 0 & 0 & {\bar{\lambda }}_{3}^{\text{D}} \end{array}\right]. \end{array}$$

Similarly, decomposing the tensor **R**_w_:

(20) $$\begin{array}{l} {\text{R}}_{\text{w}}=\xi_{\text{R}}\cdot \Lambda_{\text{R}}\cdot \xi_{\text{R}}^{\text{T}} \end{array}$$

where

(21) $$\begin{array}{l} \Lambda_{\text{R}}=\left[\begin{array}{ccc} \lambda_{1}^{\text{R}} & 0 & 0\\ 0 & \lambda_{2}^{\text{R}} & 0\\ 0 & 0 & \lambda_{3}^{\text{R}} \end{array}\right], \end{array}$$

and after rescaling the eigenvalues by their average ${\bar{\lambda }}^{\text{R}}=\frac{1}{3}\left(\lambda_{1}^{\text{R}}+\lambda_{2}^{\text{R}}+\lambda_{3}^{\text{R}}\right)$, we got ${\bar{\lambda }}_{i}^{\text{R}}=\lambda_{i}^{\text{R}}/\bar{\lambda^{\text{R}}}$ and

(22) $$\begin{array}{l} {\bar{\Lambda }}_{\text{R}}=\left[\begin{array}{ccc} {\bar{\lambda }}_{1}^{\text{R}} & 0 & 0\\ 0 & {\bar{\lambda }}_{2}^{\text{R}} & 0\\ 0 & 0 & {\bar{\lambda }}_{3}^{\text{R}} \end{array}\right]. \end{array}$$

The drug diffusion tensor was then calculated as

(23) $$\begin{array}{l} {\text{D}}_{\text{drug}}=D_{\text{cal}}\xi_{\text{D}}\cdot {\bar{\Lambda }}_{\text{D}}\cdot \xi^{{\text{T}}_{\text{D}}} \end{array}$$

where *D*_cal_ is a calibration factor and the subscripts and superscripts D indicate quantities that refer to the DT. Similarly for the other tensors:

(24) $$\begin{array}{l} {\text{K}}_{\text{drug}}=K_{\text{cal}}\xi_{\text{D}}\cdot {\bar{\Lambda }}_{\text{D}}\cdot \xi_{\text{D}}^{\text{T}} \end{array}$$

(25) $$\begin{array}{l} {\text{R}}_{\text{drug}}=D_{\text{cal}}\xi_{\text{R}}\cdot {\bar{\Lambda }}_{\text{R}}\cdot \xi_{\text{R}}^{\text{T}} \end{array}$$

(26) $$\begin{array}{l} {\text{T}}_{\text{drug}}=K_{\text{cal}}\xi_{\text{R}}\cdot {\bar{\Lambda }}_{\text{R}}\cdot \xi_{\text{R}}^{\text{T}} \end{array}$$

where the subscripts R indicate that the quantities refer to the tensor **R**. The calibration factors *D*_cal_ and *K*_cal_ are estimated from experimental data, as described in Linninger et al. (22). In accordance with Linninger et al. (22) and Stoverud et al. (45) we set $D_{\text{cal}}=10^{-12}$. We used different values for *K*_cal_ in GM and WM (22, 45). Specifically, we used $K_{\text{cal}}^{\text{WM}}=1.3\times 10^{-12}$ in WM and $K_{\text{cal}}^{\text{GM}}=0.013\times 10^{-12}$ in GM, reflecting the fact that motion of the fluid in WM can be up to 100 times faster than in GM. This procedure guaranteed that the tensors derived for the drug have the same eigenvectors as **D**_w_ and **R**_w_, respectively. Finally, we set the porosity of GM to be 0.21 and that of WM to be 0.19, as in Linninger et al. (22).

### 2.4. CFD Modeling

#### 2.4.1. Mesh

We used finite element methods to solve Equations (1–3) and (6–8). Tetrahedral meshes have been previously used to solve these equations numerically (21, 22). However, it was shown by Kim et al. (18) that using a *voxelized* mesh, namely a mesh in which the elements coincide with the voxels resulting from MR imaging, results in predictions that are in agreement with experimental results, provided that the voxels are small enough to capture the anisotropy of the microstructure that affects the motion of the drug molecules. This early work was performed on rat spinal cord. It was later shown that there is high correlation between the results obtained with a voxelized mesh and those obtained with an unstructured tetrahedral mesh for the case of drug infusion in the brain of tumor patients (19). Finally, it was shown that the predictions for the concentration when using a voxelized mesh to model drug delivery in rat hippocampus are in agreement with experimental observations (47). These studies argue, therefore, for the fact that using a voxelized mesh produces accurate results. Voxelized meshes were also used by Kim et al. (15) and Stoverud et al. (45) to model drug delivery in rat and human brain, respectively.

The main benefit of using a voxelized mesh instead of a tetrahedral one is that the physical properties of the tissue, such as the diffusivity and porosity, are assigned on a voxel-by-voxel basis rather than being interpolated from the voxels to the mesh tetrahedra. The computational cost is thus significantly reduced due to the fact that there is no need for the interpolation step in the analysis (19). For that reason, we chose to use a voxelized mesh in our analysis as well.

In our diffusion data each voxel has dimensions 1.2 × 1.2 × 1.2 mm^3^. We used the voxel nodes from the diffusion data to identify the nodes of the cubes that comprise the mesh. The mesh was subsequently generated in OpenFOAM v3.0+ (48). The mesh for the participant considered here consisted of 701, 114 cubic cells. The volume of each mesh element in our analysis was smaller than that of mesh elements previously used in the literature in human studies such as for example those used in Stoverud et al. (45) or Linninger et al. (22), resulting in more accurate allocation of WM and GM voxels. This is an important point, because, as was pointed out earlier, the drug is expected to spread faster and in larger volumes in WM as opposed to GM.

#### 2.4.2. Source Term

As has been done in other studies (18, 45), we assigned a source term representing a constant infusion rate of the fluid to one element of the mesh. This can be interpreted as a catheter opening with a diameter equal to the dimension of a mesh element, which is reasonable given that catheter openings of the order of 1 mm have been proposed as appropriate to be used in CED (13). We performed our simulations for sources located at four different points in the mesh: one located in the corpus callosum, one in the internal capsule, one in the hippocampus and one in the putamen. The corpus callosum and the internal capsule were chosen because it has been suggested that infusion in WM areas of the brain can be used to guide the drug of interest to GM areas (49), and therefore simulations of infusion in WM are necessary. The hippocampus and the putamen were chosen because they are structures of clinical significance for epilepsy and for Huntington's disease. When simulating infusion in the corpus callosum, the hippocampus, and the putamen, three different infusion rates were considered: 0.3, 1.8, and 6μl/min. These infusion rates are realistic and have been proposed, and modeled, in the CED literature. In subsequent sections, we refer to the simulations with these rates as slow, medium and fast, respectively. For the case of the slow and medium infusions, we set the pressure difference between the infusion point and the boundary surfaces of the brain to be equal to 3.5 kPa. For the case of fast infusion, we set the pressure difference equal to equal to 1.5 kPa for infusion in the corpus callosum and equal to 4.5 kPa for infusion in the hippocampus and putamen. When simulating infusion in the internal capsule, we considered a scenario that is similar to the experiments conducted by Raghavan and Brady (5), and in which the infusion took place in the internal capsule (in pig brains), starting with a rate of 2.5μl/min for 30 min followed by a rate of 5μl/min. We used those infusion rates for the same time lengths when simulating infusion in the internal capsule in our work.

#### 2.4.3. Solver

The system of Equations (1–3) and the system of Equations (6–8) were solved using modified solvers from OpenFOAM v3.0+, where the modifications were introduced to account for the tensorial nature of diffusion and convection. A weakly coupled problem was assumed (17), where the first two equations of each system were solved to obtain a steady-state solution for the velocity and pressure. The equations for the concentration were subsequently solved with the resulting fixed velocity field. The time step used to solve the transport equations was 1 s. The simulations were run for an infusion time of 72 h.

We reran the simulations with a finer mesh, to check whether changing the mesh resolution had any impact on the predicted drug concentrations. Specifically, we used OpenFOAM v3.0+ to produce a refined mesh, the cells of which have dimensions 0.6 × 0.6 × 0.6 mm^3^. This refined mesh consisted of 5, 608, 912 cells. We also used OpenFOAM v3.0+ to map the tensors (**K**, **D**, **T**, and **R**) and the porosity φ from the original mesh to the refined mesh. We reran the simulations for each of the three infusion sites and each of the two CFD models. Very good agreement was observed between the values obtained from the two different mesh element sizes, for the pressure, the velocity, and the concentration of both the R-model and the D-model.

#### 2.4.4. Comparison of Concentrations

In order to compare the concentrations derived from the two models, we calculated the fractional difference in concentration $f=\left(\frac{C_{\text{R}}-C_{\text{D}}}{C_{\text{D}}}\right)$ for all voxels, noting that this implies that *C*_R_ = (*f* + 1)*C*_D_. We also used a paired t-test for each infusion site and rate of infusion to check whether the differences in the concentration distributions within the distribution volume were statistically significant.

Since the R-model contains information about the non-Gaussianity of the diffusion process in the brain tissue, we hypothesized that, in any given location, the difference in the concentrations predicted by the two models would be correlated with the non-Gaussianity of the tissue that the fluid went through to reach that location, starting at the infusion site. For that reason, we calculated the correlations between the absolute value of the fractional differences in concentration and measures of non-Gaussianity in brain tissue. Since the effects under consideration were of second order, we calculated the correlations of the concentration fractional differences with the Difference in Covariances (DC), a measure of non-Gaussianity described in Ning et al. (39). Specifically, if $\hat{\text{r}}$ denotes the displacement of molecules that is distributed according to a Gaussian distribution G(**r**) and $\left(\hat{\text{r}}+\tilde{\text{r}}\right)$ is the displacement that is distributed according to the actual distribution P(**r**), then the Difference in Covariances, which is a measure that has been shown to account for the difference in the second order of the diffusion propagator, is (39)

(27) $$\begin{array}{l} \text{DC }\equiv \;\underset{\hat{r}}{\text{min}}\int_{R^{3}}∥\tilde{r}{∥}^{2}\text{G}\left(r\right)\text{d}r. \end{array}$$

**Figure 1:**
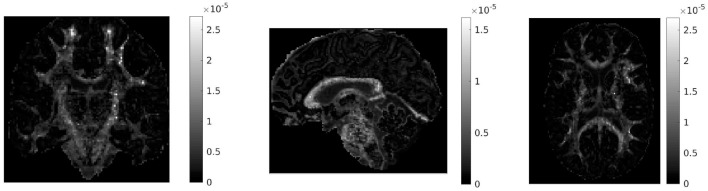
Difference in covariances (DC), a measure of second order diffusion non-Gaussianity in brain tissue, for a coronal **(left)**, sagittal **(middle)**, and axial **(right)** slice in our data. The DC is plotted on a different scale in each slice. The higher the value of DC in a voxel, the stronger the diffusion non-Gaussianity in that voxel. We notice that the DC has large values predominantly in white matter areas of the brain.

If in a given voxel diffusion is Gaussian, then $\tilde{\text{r}}=0$ and DC = 0. The higher the value of DC, the more non-Gaussian the diffusion is in that voxel. Figure 1 shows the values of DC for three representative slices in our data. WM voxels generally exhibit higher diffusion non-Gaussianity than other voxels.

**Figure 2:**
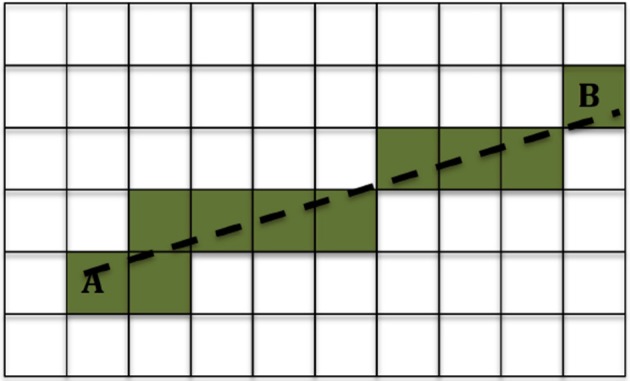
Example of the 2-dimensional Bresenham algorithm (50) for identifying the squares for the line that connects square A to square B. The shaded squares are the ones chosen.

We used the method described in Ning et al. (39) to calculate the DC in each voxel. Then for each location of interest we used the 3-dimensional Bresenham algorithm (50) to identify the sequence of voxels connecting the infusion site to the location of interest, and summed the DCs of the voxels in that sequence. A drawing showing the Bresenham algorithm for a 2-dimensional case is shown in Figure 2; the algorithm is similar in 3D. The sequence of voxels given by the Bresenham algorith is, of course, not the exact path that the fluid follows while moving from the infusion site to the location of interest. However that exact path cannot be known with certainty, and in fact different fluid molecules are bound to follow different paths to reach a given location in the brain. The sum of DCs that we used gives an approximate, representative measure of the non-Gaussianity that the fluid molecules encounter while traveling from the infusion site to the location of interest.

## 3. Results

### 3.1. Comparison of the Tensors D_w_ and R_w_

The difference between the two CFD models presented in this work comes from the use of the tensor **R** instead of **D** in modeling the movement of the fluid in the brain tissue. As explained earlier, we first performed a detailed comparison of the properties of the two tensors, to the extent that we expect these properties to affect the results of the fluid dynamics simulations.

**Figure 3:**
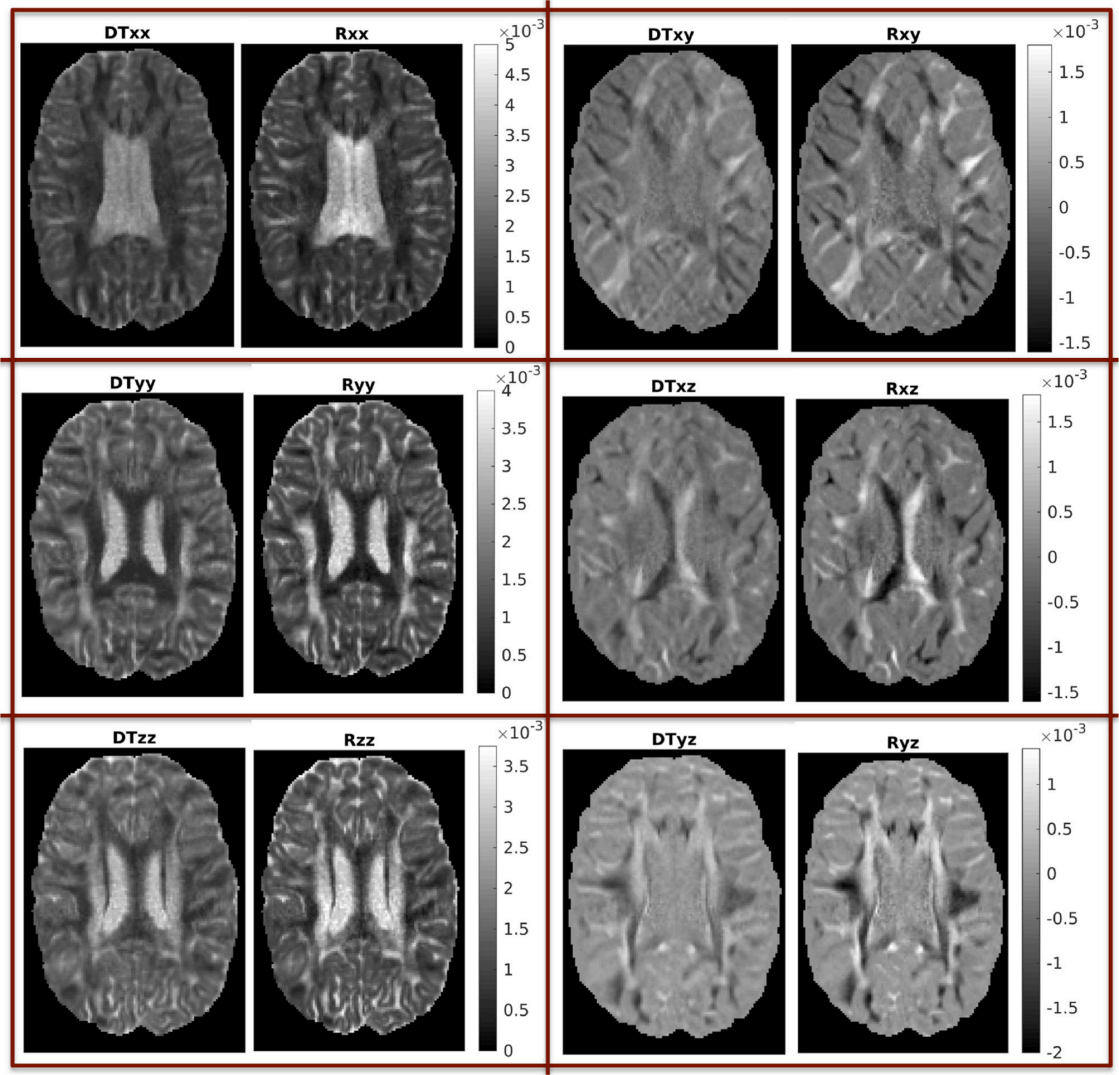
Components of the two tensors for axial slice 70 (units of mm^2^/s). For ease of comparison, corresponding components were plotted in the same intensity scale.

In Figure 3 we show the components of each tensor for one axial slice. For ease of comparison, corresponding components have been plotted in the same intensity scale. The three diagonal components assume comparable values for both tensors, with the components of **R**_w_ assuming slightly higher values in WM areas, a fact that reflects the highly non-Gaussian nature of diffusion in those brain areas. The non-diagonal components of **R**_w_ have values in a wider range than those of **D**_w_, so that their maxima are larger than those of the corresponding c components of **D**_w_, and their minima are smaller than those of the corresponding components of **D**_w_.

**Figure 4:**
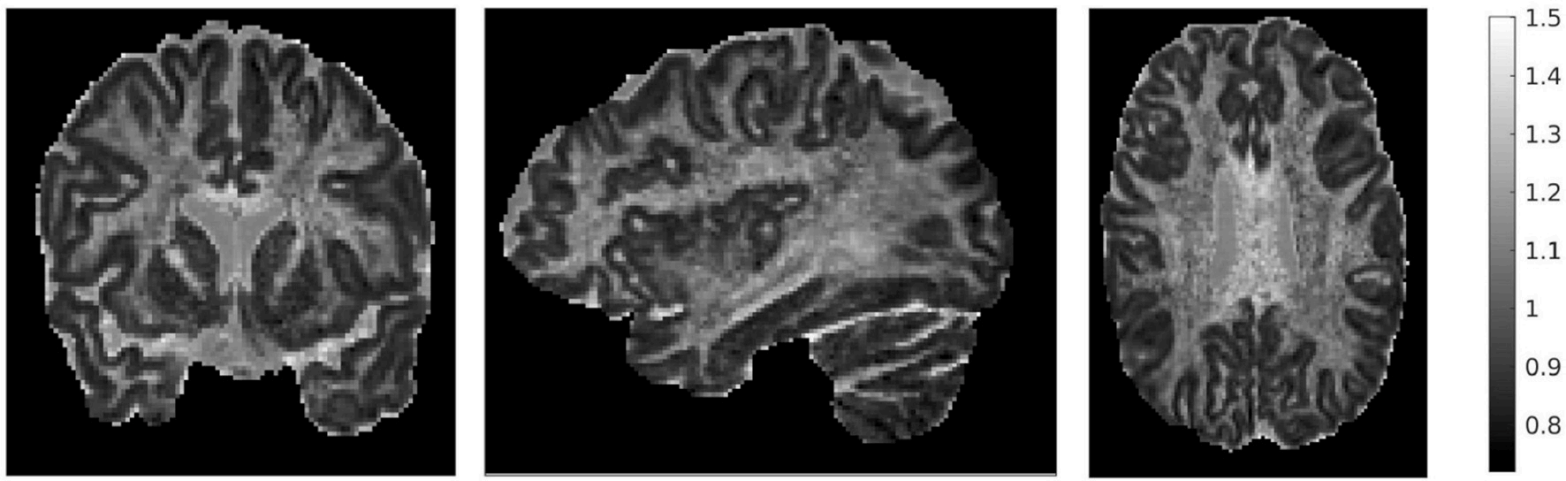
Ratio of traces of the two tensors $\frac{\text{Tr}\left({\text{R}}_{\text{w}}\right)}{\text{Tr}\left({\text{D}}_{\text{w}}\right)}$, for three representative slices: coronal slice 76 **(left)**, sagittal slice 67 **(middle)**, and axial slice 71 **(right)**. The ratio of the traces is around 1, with lower values in the cortical areas and higher values in areas of higher anisotropy.

We also mentioned earlier that we calculated the ratio of the traces of the two tensors. That ratio is shown in Figure 4, for three different brain slices. The ratio is around the value of 1, with cortical structures exhibiting values that are slightly lower, and highly anisotropic structures exhibiting values that are slightly higher. It also has higher values in the dura mater surrounding the cerebellum, which consists of fibrous tissue in a fanning arrangement.

**Figure 5:**
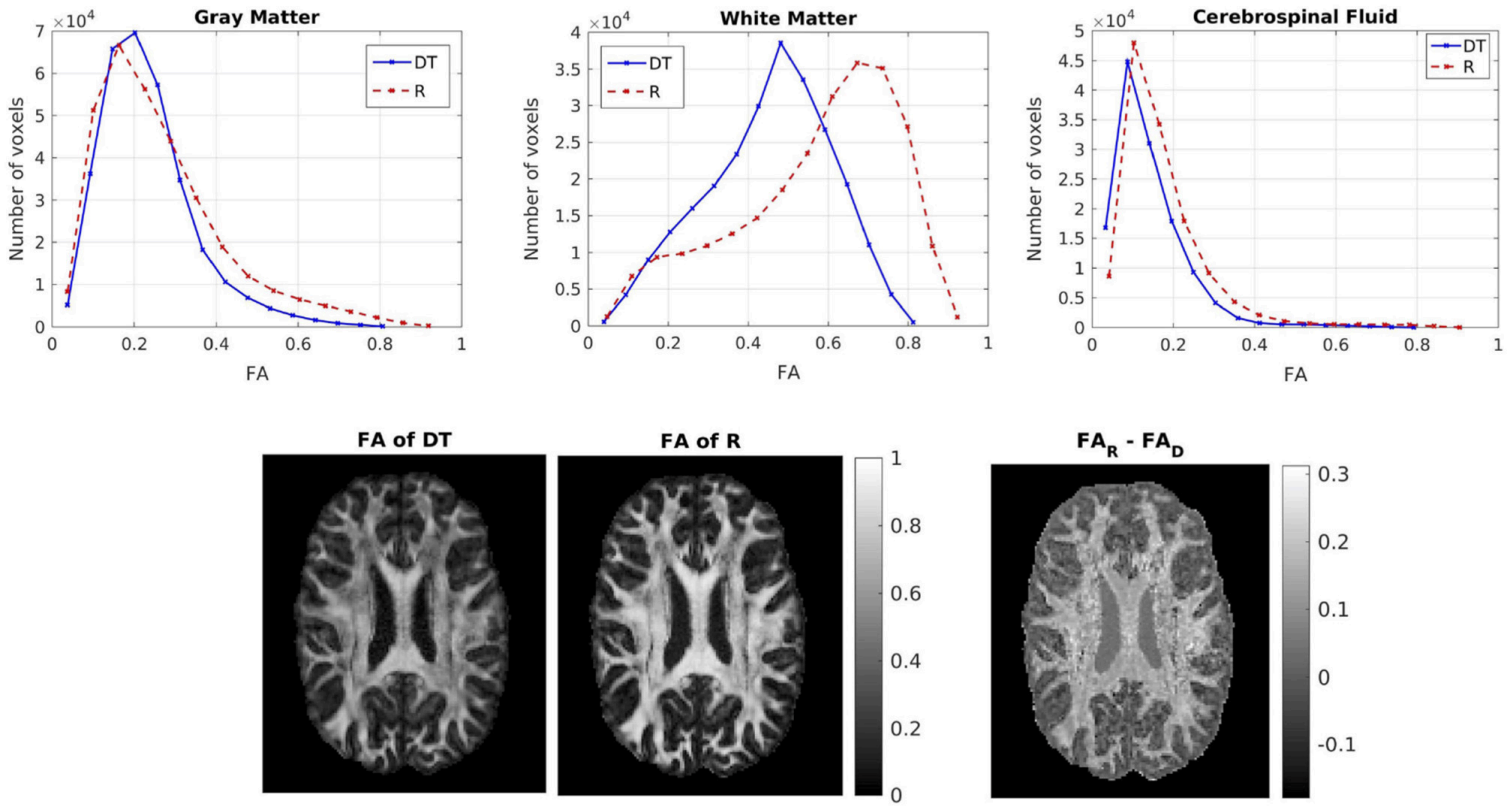
**(Top)** Histogram of the FA of the two tensors in voxels identified as GM (left), WM (center), and CSF (right). The solid lines represent **D**_w_ and the dashed lines represent **R**_w_. **(Bottom)** FA of D_w_ (left), FA of R_w_ (center), difference of the FAs (right) for axial slice 70. The FAs of the two tensors are plotted on the same scale.

We then compared the FAs of the two tensors. Figure 5 shows the distribution of the FAs of the two tensors, for GM, WM, and CSF separately. For all three compartments, the distribution of the FA of the tensor **R**_w_ is shifted to higher values with respect to that of the tensor **D**_w_, with the larger differences appearing in WM. We performed a paired t-test to compare the distributions of the FAs of the two tensors, separately for GM, WM and CSF. In all three cases the FA of **R**_w_ was statistically significantly higher than that of **D**_w_ (*p*-value < 10^−10^). To further demonstrate the differences in FA, we show in the lower part of Figure 5 the FA of **D**_w_ (left panel, d) and that of **R**_w_ (middle panel, e) for an axial slice, while the right panel (f) shows the difference between the two anisotropies for the same slice. The larger differences in anisotropy appear mainly in the white matter areas.

**Figure 6:**
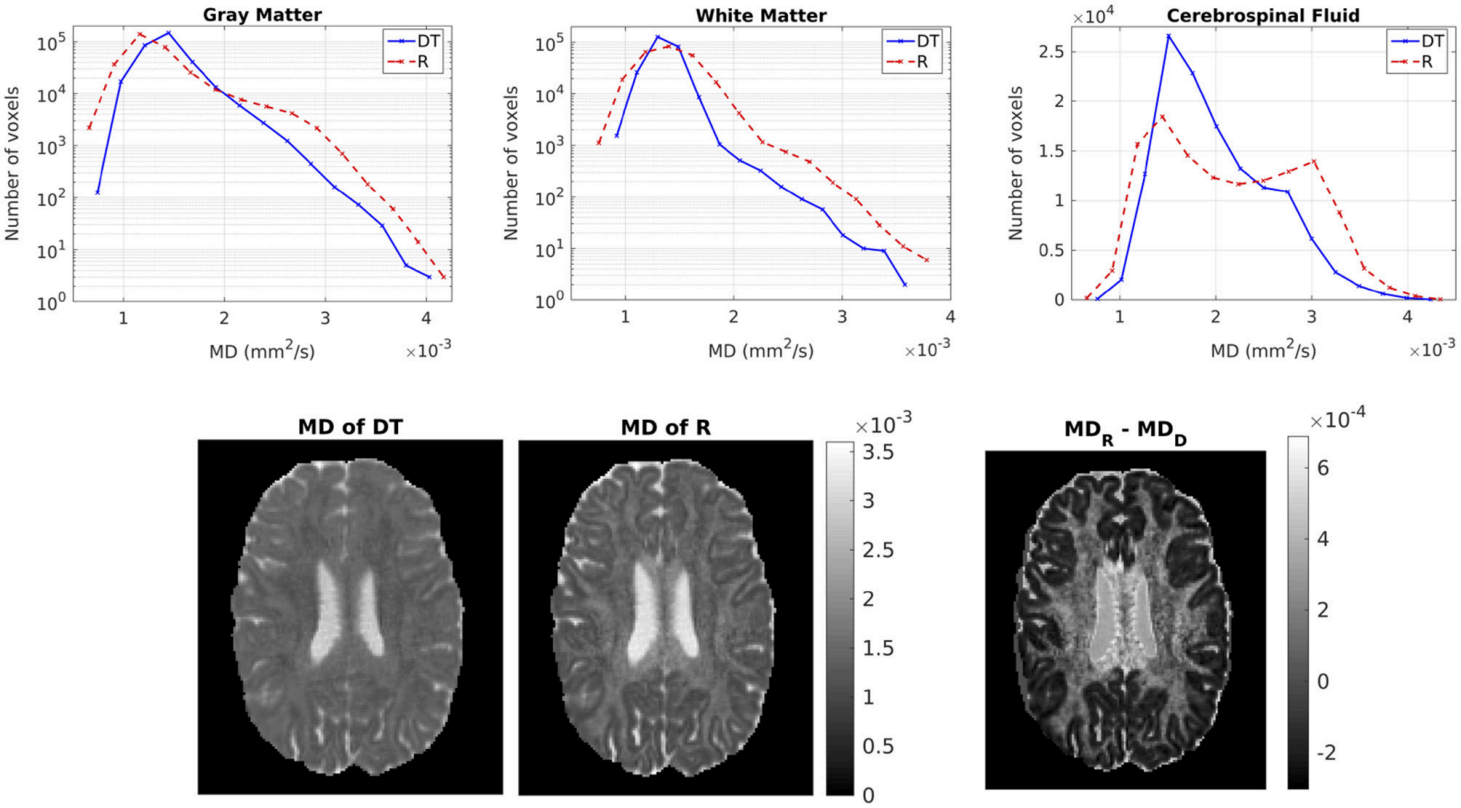
**(Top)** Histogram of the MD of the two tensors in voxels identified as GM (left), WM (center), and CSF (right). The solid lines represent **D**_w_ and the dashed lines represent **R**_w_. **(Bottom)** MD (in mm^2^/s) of D_w_ (left), MD of R_w_ (center), difference of MDs (right) for axial slice 70. The MDs of the two tensors are plotted on the same scale.

Figure 6 shows the distribution of the MDs of the two tensors, for GM, WM and CSF separately. Notice the logarithmic scale used for the y-axis for the distributions for GM and WM. For all three compartments, the distribution of the MD for **R**_w_ is broader than that for **D**_w_. We performed a paired t-test to compare the distributions of the MDs of the two tensors, separately for GM, WM and CSF. In all three cases, the MD distribution for **R**_w_ was statistically significantly different to that of **D**_w_ (*p*-value < 10^−10^). To further demonstrate the differences in MD, we show in the lower part of Figure 6 the MD of **D**_w_ (left panel, d) and that of **R**_w_ (middle panel, e) for an axial slice, while the right panel (f) shows the difference between the two MDs for the same slice.

Finally, we calculated the mean of the FA and the MD for both tensors separately in each compartment (GM, WM, and CSF), and the results are given in Table 2.

**Table 2:** Mean FA and MD for GM, WM, and CSF for **D**_w_ and for **R**_w_.

	**Mean FA of *D*_w_**	**Mean FA of *R*_w_**	**Mean MD of *D*_w_**	**Mean MD of *R*_w_**
GM	0.233	0.265	1.43 × 10^−3^	1.34 × 10^−3^
WM	0.451	0.569	1.36 × 10^−3^	1.41 × 10^−3^
CSF	0.140	0.175	2.00 × 10^−3^	2.17 × 10^−3^

### 3.2. Predictions of Drug Concentration

**Figure 7:**
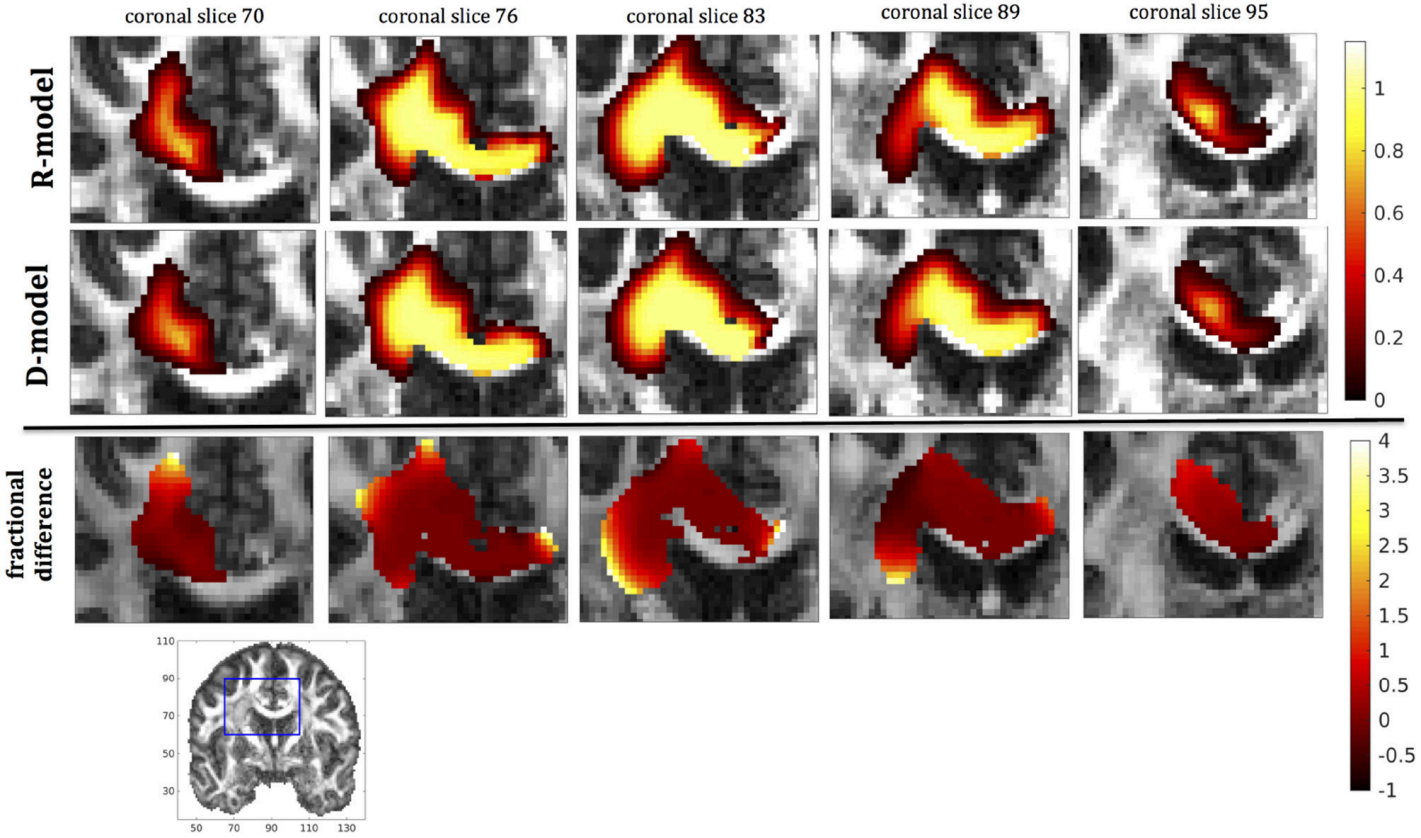
Predicted concentrations for fast infusion in the corpus callosum. **(Top)** R-model prediction for 5 coronal slices, **(Middle)** D-model prediction for the same slices, **(Bottom)** fractional difference in concentrations $\left(\frac{C_{\text{R}}-C_{\text{D}}}{C_{\text{D}}}\right)$. The same scale has been used to plot the concentration distributions for the R- and the D- model. The FA image at the bottom shows in blue the part of the brain slices shown above.

**Figure 8:**
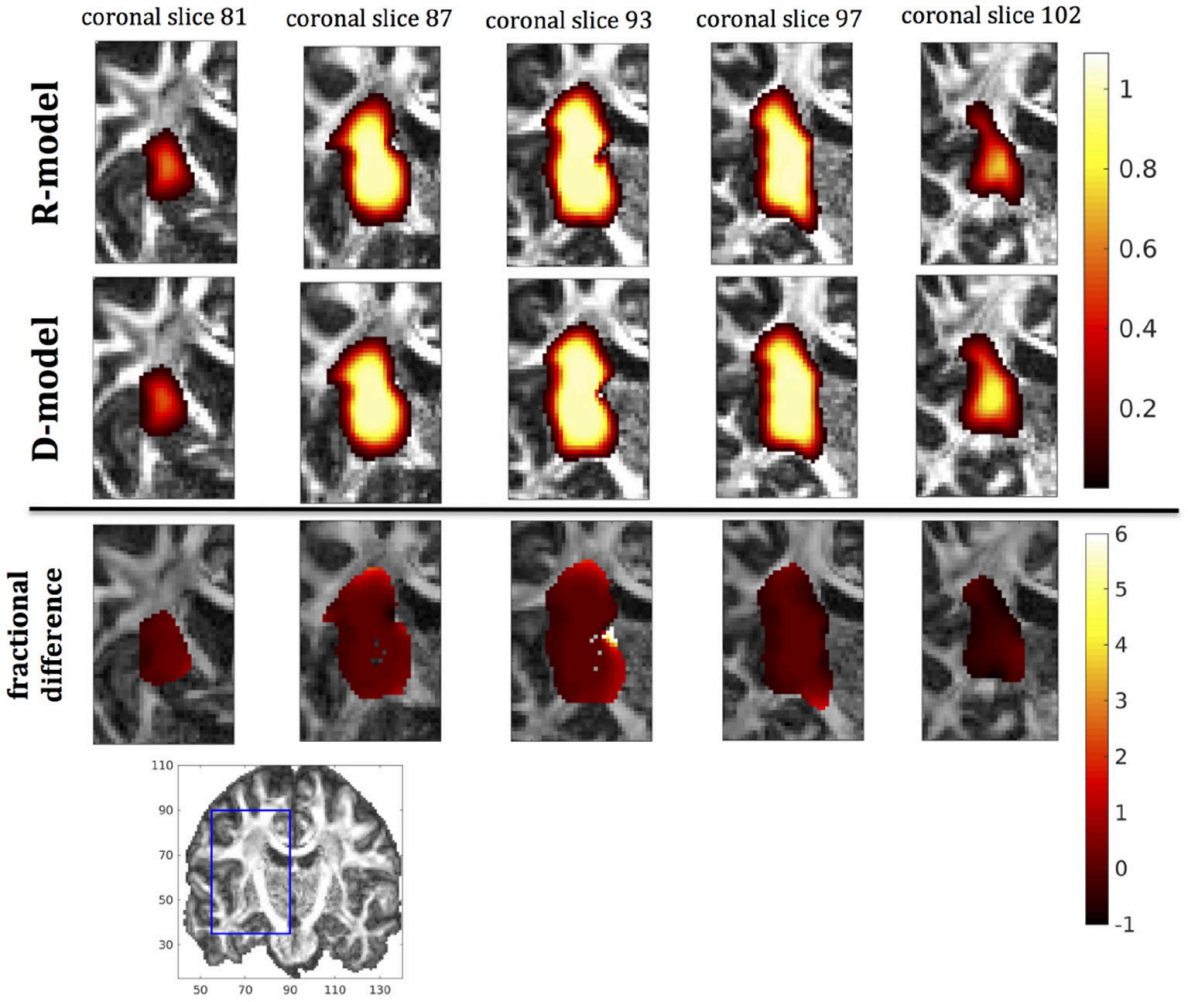
Predicted concentrations for infusion in the internal capsule. **(Top)** R-model prediction for 5 coronal slices, **(Middle)** D-model prediction for the same slices, **(Bottom)** fractional difference in concentrations $\left(\frac{C_{\text{R}}-C_{\text{D}}}{C_{\text{D}}}\right)$. The same scale has been used to plot the concentration distributions for the R- and the D- model. The FA image at the bottom shows in blue the part of the brain slices shown above.

**Figure 9:**
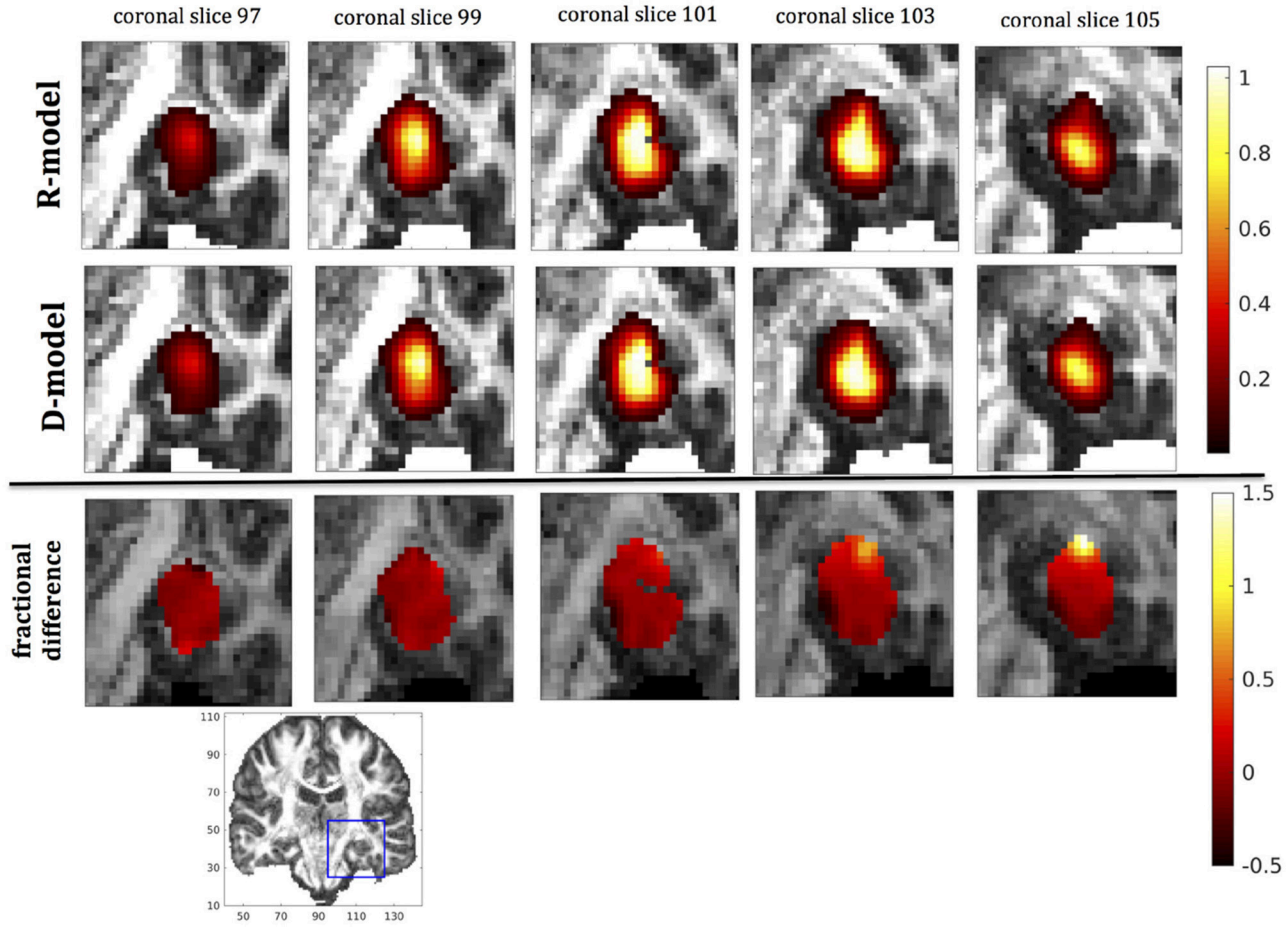
Predicted concentrations for fast infusion in the hippocampus. **(Top)** R-model prediction for 5 coronal slices, **(Middle)** D-model prediction for the same slices, **(Bottom)** fractional difference in concentrations $\left(\frac{C_{\text{R}}-C_{\text{D}}}{C_{\text{D}}}\right)$. The same scale has been used to plot the concentration distributions for the R- and the D- model. The FA image at the bottom shows in blue the part of the brain slices shown above.

**Figure 10:**
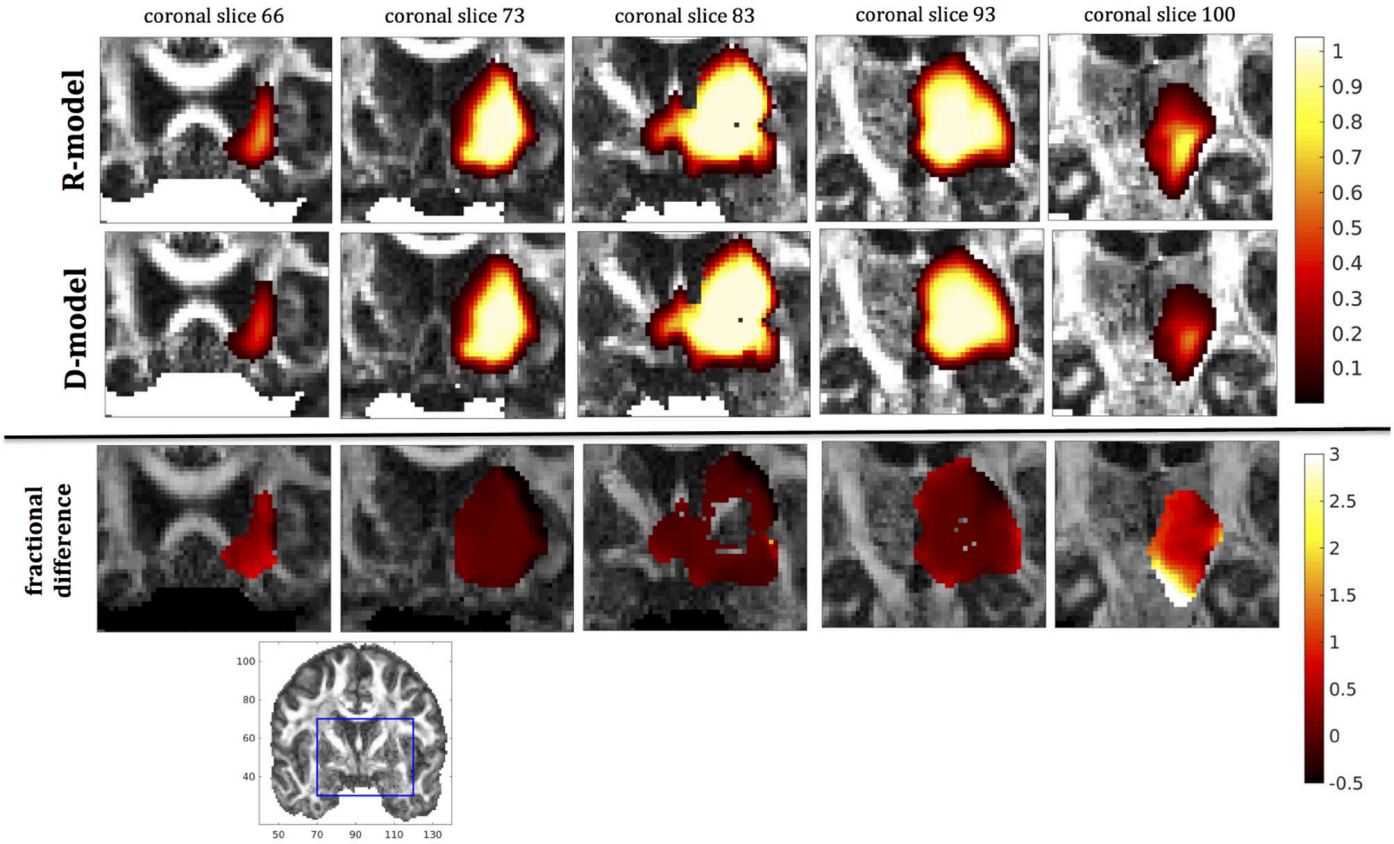
Predicted concentrations for fast infusion in the putamen. **(Top)** R-model prediction for 5 coronal slices, **(Middle)** D-model prediction for the same slices, **(Bottom)** fractional difference in concentrations $\left(\frac{C_{\text{R}}-C_{\text{D}}}{C_{\text{D}}}\right)$. The same scale has been used to plot the concentration distributions for the R- and the D- model. The FA image at the bottom shows in blue the part of the brain slices shown above.

In this section we present the results of our simulations. Figures 7–10 show the concentrations predicted by the two CFD models for 72 h of infusion time, for fast infusion in the corpus callosum, the internal capsule, the hippocampus and the putamen, respectively. In each Figure, the top row shows the concentration predicted by the R-model for five representative coronal slices. For comparison, we also show the concentration predicted by the D-model for the same coronal slices in the middle row. The same scale was used to plot the concentration distributions for both models for each infusion site. In the bottom row of each Figure, we show the fractional difference in concentration $f=\left(\frac{C_{\text{R}}-C_{\text{D}}}{C_{\text{D}}}\right)$ for the same coronal slices. The predictions of the R-model were in very good agreement with observations existing in the literature. Specifically, the spread of the drug in gray matter was isotropic, while the spread in white matter areas was anisotropic and followed the anisotropy of the underlying fibers. In all cases, we observed an extended plateau of constant concentration spanning a distance of a few cm in each direction, followed by a sharp drop in the concentration. The results were similar for the cases of medium and slow infusion.

It was proven in Equation (12) that there are differences between the drug concentration predicted by the R-model and that predicted by the D-model, and they depend on the duration of infusion, the location of the infusion site and pressure and velocity of the drug. Our simulations allow a quantification of those differences. Observing the distributions predicted by the two models in Figures 7–10, some differences could be discerned, relating to both the volume covered by the drug and the concentration of the drug in each brain location. For a more detailed understanding of the differences, we focused on the plots of the fractional differences in the above Figures. The first observation was that the fractional differences in concentration were relatively small for the central part of the plateau of constant concentration predicted by the two models. Specifically, they reached values of ±0.1 for infusion in the corpus callosum, ±0.15 for infusion in the internal capsule, ±0.04 for infusion in the hippocampus, and ±0.07 for infusion in the putamen. The differences became larger for the outer edges of the plateau, for example they reached values of ±0.2 for infusion in the corpus callosum, indicating that for some parts of the plateau, the expected drug concentration can be up to 20% higher than that predicted when diffusion non-Gaussianity is not taken into account. In addition to that, the values of the fractional difference were larger for the front of the spread of the drug. Specifically, for the case of the corpus callosum and the internal capsule, they routinely reached values of up to 7 in voxels where the concentration was in the range of 5–10% of the maximum concentration, indicating that the concentration predictions can be incorrect by a large factor if diffusion non-Gaussianity is not taken into account. This is important because it implies that including the effects of non-Gaussianity in the analysis results in different conclusions when assessing which brain locations are reached by the drug, and with what concentration.

**Figure 11:**
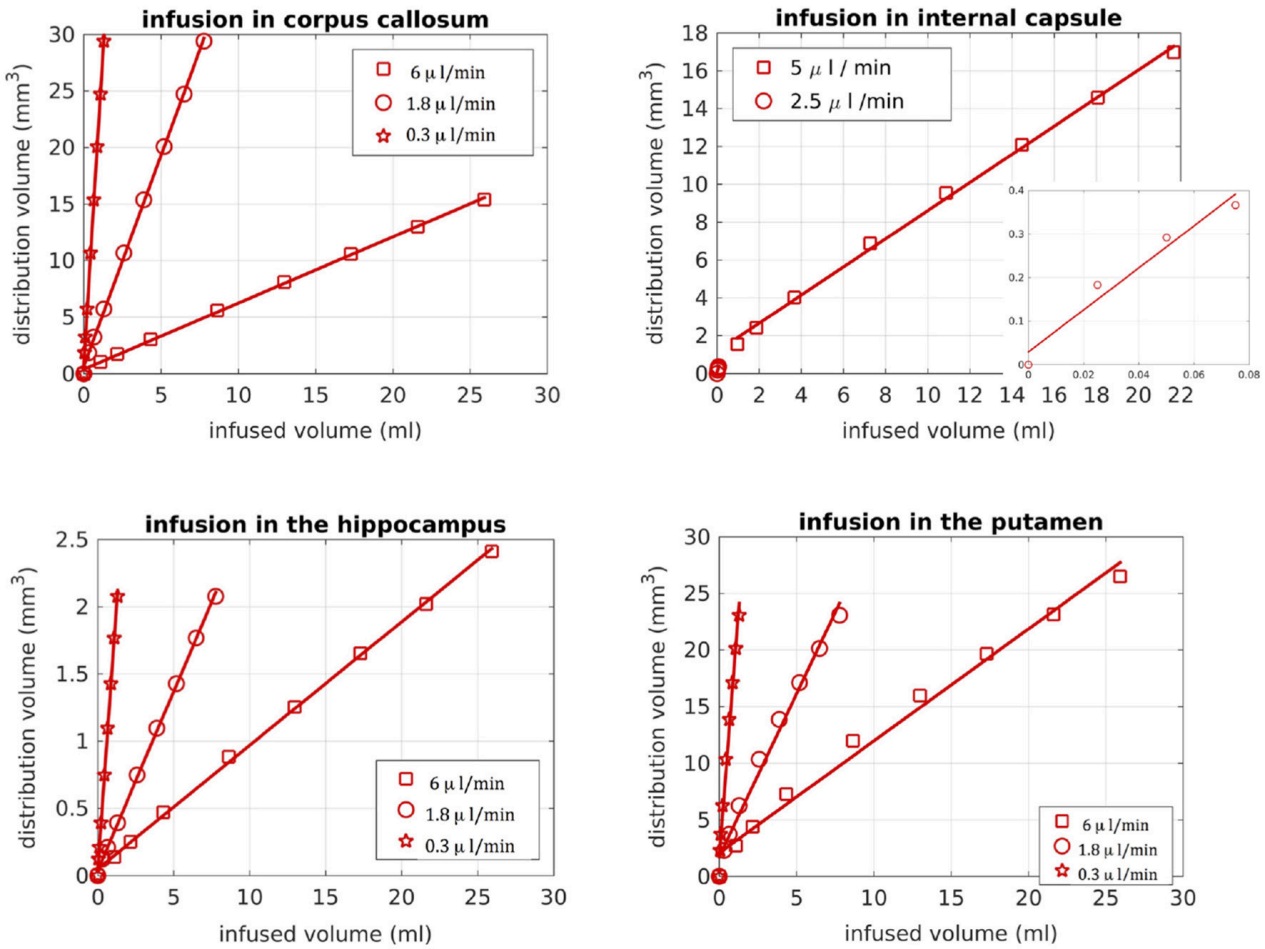
Distribution volume vs. infused volume, for the R-model. The relationship is linear for all infusion sites.

We also looked into the distribution volume of the drug. Different studies have used different definitions for the distribution volume of the drug. For example, in Kim et al. (15) and Linninger et al. (22) the distribution volume was defined as the volume of the mesh elements in which the drug concentration was at least 5% of the maximum concentration, while in Sarntinoranont et al. (17), Kim et al. (18), and Magdoom et al. (20) that threshold was 15%. In our work, we defined the distribution volume as the volume of the voxels in which the drug concentration reached 5% or higher of the maximum concentration. Figure 11 shows the distribution volumes resulting from our simulations with the R-model for the 4 infusion sites for different volumes of the infused drug. As expected, the relationship is linear in all cases. Additionally, higher infusion rates resulted in lower distribution volumes. This result is in agreement with the experimental observations of Magdoom et al. (20).

As previously mentioned, we used a paired t-test for each infusion site and rate to check whether the differences in the concentration distributions within the distribution volume were statistically significant. For infusion in the corpus callosum, the differences reached statistical significance (*p*-value of *t-*test < 0.01) at under 3 h of infusion for slow and medium infusions and at 6 h of infusion for fast infusion, increasing in magnitude for later times. For infusion in the internal capsule, the differences reached statistical significance at under 1 h of infusion. For infusion in the putamen, the differences reached statistical significance at 3 h of infusion for the cases of slow and medium infusions and at 1 h for the case of fast infusion, also increasing in magnitude for later times. For infusion in the hippocampus, the observed differences reached statistical significance at under 60 h for the cases of slow and medium infusion and at 48 h for the case of fast infusion.

**Figure 12:**
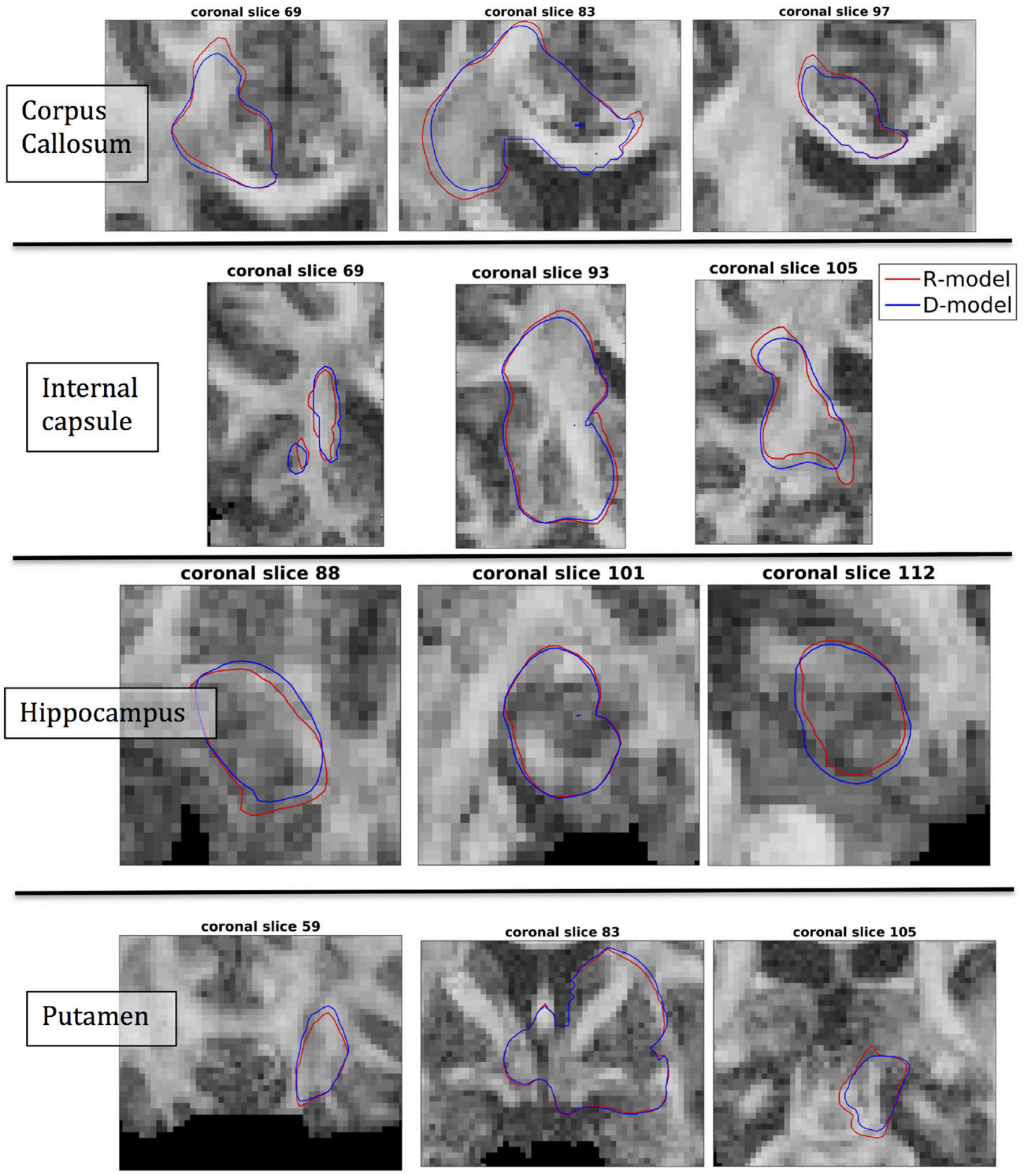
Distribution volumes (i.e., volumes where the drug reaches a concentration that is at least 5% of the maximum concentration) predicted by the R-model (red curves) and by the D-model (blue curves), for the 4 infusion sites used in our simulations. Accounting for diffusion non-Gaussianity results in distribution volumes that are less smooth and more anisotropically defined, following the anisotropy of the underlying tissue.

The fact that the tensor **R** has different eigenvectors to the tensor **D** means that the R-model would tend to channel the fluid along different directions in principle, which would then reach different brain structures. This is important because it has direct implications on the recommendations given to clinicians for the practical implementation of CED, regarding the positioning of the catheters and the infusion pressure and flow rate. We plotted the edge of the distribution volume for the two different models on an FA map of the brain for a few representative coronal slices, and compared the structures that the drug reaches based on each model, for the 4 infusion sites. The results are shown on Figure 12. The largest differences between the voxels reached by the drug appear for the case of infusion in white matter, namely the corpus callosum and the internal capsule. The differences were similar to the discrepancies between predictions derived by the D-model and experimental observations reported by Raghavan et al. (5) for infusion in the internal capsule of pig brain. Specifically, for infusion in white matter areas, the distributions predicted when diffusion non-Gaussianity was disregarded were smoother and less sharply defined than those predicted when it was taken into account.

As we explained earlier, we looked into possible correlations of the differences in concentration with the DC of the brain tissue. The correlation coefficients between the absolute value of the fractional difference in the concentrations and the sum of DCs along the Bresenham line for the four infusion sites are given in Table 3, for three different time points of our simulations. All correlations were positive and highly statistically significant (*p*-value < 10^−10^), and persisted over time. This indicates that the higher the sum of the DCs in the tissue between the infusion site and the location of interest, the larger the differences in the concentrations predicted by the two models.

**Table 3:** Correlation coefficients between the fractional difference in concentration and the non-Gaussianity the sum of the DCs along the Bresenham line that connects the infusion voxel and the voxel of interest, for infusion in the hippocampus and the putamen, for all three infusion rates and for 6 different infusion times.

	**Fast**			**Medium**			**Slow**		

	**24 h**	**48 h**	**72 h**	**24 h**	**48 h**	**72 h**	**24 h**	**48 h**	**72 h**
Corpus callosum	0.23	0.23	0.18	0.18	0.12	0.12	0.18	0.12	0.12
Internal capsule	0.38	0.35	0.30						
Hippocampus	0.45	0.43	0.43	0.45	0.40	0.45	0.43	0.40	0.45
Putamen	0.40	0.48	0.54	0.39	0.48	0.55	0.39	0.48	0.55

*The p-values for all correlations were lower than 10^−10^, indicating highly statistically significant correlations*.

We also examined the correlations between the absolute value of the fractional difference in the concentrations and the sum of the FAs of the voxels in the Bresenham line, however these correlations, even though statistically significant in most cases, were weaker than the correlations with the sum of the DCs and for that reason we do not dwell on them here.

## 4. Discussion

We presented a theoretical framework and the corresponding CFD model for modeling convection-enhanced drug delivery to the human brain, at an individual-participant level. We also used high-resolution human diffusion MRI data to perform simulations of CED with our CFD model and with the CFD model that has been previously used in the literature. Our novel model, abbreviated as R-model, relies on the diffusion propagator, while the previous model, abbreviated as D-model, relies on the DT. The key development in our work is that our model does not assume Gaussian diffusion in brain tissue, but rather employs the MR-measured diffusion probability along all directions, therefore incorporating the second-order effects of restriction and hindrance on the motion of the drug macromolecules.

Both the validity of our model and the need for including diffusion non-Gaussianity when modeling CED are supported by experimental results already present in the literature. Firstly, the R-model gave results for the drug distribution that are in agreement with experimental observations, with the drug spreading isotropically in gray matter areas, while exhibiting an anisotropic distribution that follows the direction of the underlying fibers in white matter areas. Additionally, the R-model predicted drug distributions in white matter areas that are more anisotropic and less smooth than those predicted by the D-model. Raghavan et al. (5) reported that their simulations, using the DT, predicted distributions that were smoother and less anisotropic compared to the observed distributions, when performing infusion in the internal capsule of pig brains. Our simulations using the tensor **R**, which represents the tissue diffusion anisotropy more accurately than the DT, resulted in predictions that exhibit exactly the behavior observed experimentally by Raghavan et al. (5).

Additionally, there are other instances in the literature where statistically significant differences have been reported between experimentally observed drug distributions and those predicted by the model that uses the DT, and where taking into account the effects of diffusion non-Gaussianity could improve the predictions. For example, in the study presented by Kim et al. (47), statistically significant differences were observed between the predicted and the observed drug distributions for infusion in rat hippocampus. The differences were up to 20.7% and related to the areas that were predicted to be covered by the fluid compared to those that were actually covered. Using the R-model could mitigate some of the differences. Additionally, Kim et al. (18) compared their computational predictions of the distribution volume with the experimental observations of Wood et al. (51), for infusion in rat spinal cord, and found statistically significant differences between the two. Even though the authors offered some reasons for the differences, it is possible that predictions could be improved if the R-model were used and the diffusion non-Gaussianity that is known to be present in the highly anisotropic spinal cord were properly accounted for in the model.

It is important to keep in mind that our analysis was performed on data collected from a healthy participant. Pathological brain tissue, which will ultimately be the target of CED treatment, could be even more affected by the effects of diffusion non-Gaussianity. We showed in our analysis that the higher the sum of the DCs in the tissue between the infusion site and a location of interest, the larger the difference between the concentrations predicted be the two models. Studies of diseased brain that exist in the literature, however, have not investigated the DC in diseased brain but rather the FA of **D** and how that changes as a result of disease. Both the FA and the DC relate to the anisotropy of the brain tissue and we therefore expect that they will be correlated. We calculated the correlation coefficient between FA and DC in the brain of our healthy participant and found it to be 0.54 (*p*-value < 10^−10^), indicating that in general the higher the DC is in a voxel, the higher the FA is as well. A recent meta-analysis of data from HD patients (52) showed that there is elevated FA in the caudate and the putamen in symptomatic HD patients compared to controls. Therefore, the effect of taking non-Gaussianity into account could be larger in those patients than our simulations for infusion in the putamen of the healthy participant indicate. Similar conclusions apply in patients with temporal lobe epilepsy, who were shown to have altered microstructure in the hippocampus compared to age-matched controls, with a higher FA in the dentate gyrus than age-matched controls (53). Additionally, in a recent study of 21 PD patients scanned at 3T, the PD patients were found to have an average FA of about 0.6 in the substantia nigra and of about 0.5 in the globus pallidus (54), which were again higher than the average FA in the same brain structures of healthy controls. This, again, indicates that it will be necessary to correctly account for the effects of diffusion non-Gaussianity when simulating CED for those patients. Finally, it has been shown that, in the case of ischemic stroke, there is a decrease in the extracellular space of the affected tissue which can be up to 75% (11). This means more restriction for the motion of the drug molecules than expected in healthy subjects, which makes it necessary for the effects of non-Gaussianity to be taken into account when modeling CED.

Considering the suggested future directions for CED, it becomes evident that the effects of diffusion non-Gaussianity need to be properly accounted for in CFD models. For example, it has been proposed (10) that a potential future avenue for more effective CED therapies, in particular for brain tumors, could be to use infusion for long times, comparable to the times in the simulations performed in our work. Longer infusion times relate to larger distribution volumes and therefore larger values for the sum of the DCs between the infusion point and the various structures that are covered by the drug. The positive correlations between the sum of the DCs and the fractional differences in concentration observed in our work imply that, especially for the structures that are furthest from the infusion location, not accounting for diffusion non-Gaussianity could lead to significant over- or under-estimation of the drug concentration in those structures. It has also been proposed that infusion in WM structures could be used to guide the therapeutic agents to GM structures faster. The effects of diffusion non-Gaussianity on the distribution of the drug were more pronounced in the case of infusion in WM structures (such as the corpus callosum and the internal capsule examined in our work), and for these cases it would be necessary for those effects to be appropriately taken into account in the computational simulations by using the R-model rather that the D-model. Another proposed area of future development for CED relates to considering the size and shape of the therapeutic macromolecules (13). The larger the molecules that have to move in the small extracellular space in the brain tissue, the stronger they will feel the effects of restriction, and the larger the error made by using the D-model to predict the drug distributions through CED. Using a model that properly accounts for diffusion non-Gaussianity will be necessary in that case.

It is worth pointing out that the resolution used in our diffusion scans, namely (1.2 mm)^3^, is much finer than that routinely used when collecting human diffusion MRI data, which is usually (2 mm)^3^. As a result, the volume of each voxel, and therefore each mesh element, in our study is 4.6 times finer than otherwise, resulting in the tissue in each voxel being more accurately assigned as GM, WM, or CSF, and thus rendering the simulations more accurate than otherwise. Crucially, the scanning time required to achieve this resolution in the Connectom scanner at CUBRIC was, as previously mentioned, 19 min, which is well tolerated by most participants, including vulnerable populations such as patients. We should mention, however, that this high resolution, although desirable, is not necessarily essential for accurate simulations. It has been shown in the literature that CFD simulations using diffusion MRI scans of lower resolution (22, 45) can yield predictions that are, at the very least, in very good qualitative agreement with experimental observations. More research in this direction is needed, so that the optimal resolution that minimizes the scan time while providing the details of brain tissue microstructure needed to produce accurate simulations is identified.

Our work has a few limitations. Firstly, we did not include in our model the effects of clearance or metabolism of the drug in the brain. Such terms are discussed in some papers such as (22), however they are not known with certainty and in any case they would be identical in the R-model and the D-model and therefore would not change the results of our analysis.

Additionally, we modeled the catheter as a point source, which is a simplification of the actual situation. However, the effect of this simplification will be the same for both models and, again, does not affect the conclusions of our work. In fact, there are some proposed methods of drug delivery via MRI-guided canulas (55), for which no catheter is needed and for which the approach used in this and other works to model the source term is very realistic. Despite that, in future work related to this project, we will incorporate the catheter in the simulations, which, in addition to making the simulations more realistic, will allow us to investigate effects such as backflow.

In our CFD model, and, to the best of our knowledge, in the majority of the models that are based on the diffusion tensor (15–18, 21, 22), the brain tissue is assumed to be a rigid structure. This is, however, an approximation and is discussed by Stoverud et al. (45) and references therein. In the future, models that use the diffusion displacement covariance tensor should treat the brain tissue as an elastic medium.

Finally, the main limitation of this work is the lack of experimental validation of the results of the simulations. Our goal was to present a theoretical model that predicts drug distributions during CED and which has the potential to address some of the limitations of other theoretical models previously presented in the literature. We presented evidence from the literature to show that our theoretical model's predictions are in agreement with existing experimental results, for example as regards to the flow of the drug in brain structures of various anisotropies, as well as the drug distribution volume. We also presented evidence that shows that accounting for diffusion non-Gaussianity, as in our R-model, results in drug distributions that exhibit the anisotropy which is observed during infusion in animal brains and which is missing from the predictions of the D-model. Despite that, experimental validation is desirable. For that reason, we will perform infusion of gadolinium on phantoms that mimic the human brain structure, and on animals, and monitor its spread using MR imaging.

## Author Contributions

EM: conception of the project, theoretical analysis, development of the fluid dynamics code, diffusion preprocessing, simulations and statistical analysis of the results, writing the manuscript. SUR: scanning, contributions to the diffusion preprocessing pipeline. GP: contributions to the diffusion preprocessing pipeline. WG and DJ critical review of the manuscript, guidance on the project.

### Conflict of Interest Statement

The authors declare that the research was conducted in the absence of any commercial or financial relationships that could be construed as a potential conflict of interest.

## Appendices

### A. Derivation of the Transport Equation

The starting point of the new framework that describes the motion of the molecules of a fluid in a porous medium is the Master Equation described by (56), and independently by (57):

(A1) $$\begin{array}{l} \frac{\partial C\left(\text{r},t\right)}{\partial t}=-\underset{{\text{r}}^{\prime }}{\sum }w\left({\text{r}}^{\prime },\text{r}\right)C\left(\text{r},t\right)+\underset{{\text{r}}^{\prime }}{\sum }w\left(\text{r},{\text{r}}^{\prime }\right)C\left({\text{r}}^{\prime },t\right). \end{array}$$

In this equation, *w* is the transition rate of the fluid molecules from position **r** to **r**′ in a given time. This Master Equation expresses the conservation of mass, and describes the rate of change of the concentration at point **r** as the change due to all particles moving away from point **r** toward all neighboring points **r**′ (thus the minus sign in the first term of the right-hand side of the equation) and that due to all particles moving toward point **r** from all neighboring points **r**′. The simplicity and fundamentality of Equation (A1), as well as the fact that it can encompass phenomena in a wide range of spatial and temporal scales, has resulted in its extensive use in physics, chemistry, and fluid dynamics problems.

In order to proceed with Equation (A1) in our fluid dynamics scenario we need to know *w*(**r**, **r**′). This requires detailed knowledge of the background in which the fluid particles move, which, in the case of CED, includes characterization of the microstructure of the brain tissue.

The drug concentration is assumed to be slowly varying over finite length scales, which is a valid assumption based on experimental results for CED (14, 16, 21). A Taylor series expansion for the concentration at point **r**′ (keeping terms up to second order in the distance between the points **r** and **r**′) gives:

(A3) $$\begin{array}{l} C(r^{\prime },t)\approx C(r,t)+(r^{\prime }-r)\cdot \nabla C(r,t)\\ \;+\frac{1}{2}(r^{\prime }-r)\cdot (r^{\prime }-r):\nabla \nabla C(r,t) \end{array}$$

where the dyadic symbol : denotes the tensor product. Substituting (A2) into (A1), a partial differential equation for the concentration is derived:

(A3) $$\begin{array}{l} \begin{array}{c} \frac{\partial C\left(\text{r},t\right)}{\partial t}=\underset{{\text{r}}^{\prime }}{\sum }\left[w\left(\text{r},{\text{r}}^{\prime }\right)-w\left({\text{r}}^{\prime },\text{r}\right)\right]C\left(\text{r},t\right)\\ +\underset{{\text{r}}^{\prime }}{\sum }w\left(\text{r},{\text{r}}^{\prime }\right)\left({\text{r}}^{\prime }-\text{r}\right)\cdot \nabla C\left(\text{r},t\right)\\ +\underset{{\text{r}}^{\prime }}{\sum }w\left(\text{r},{\text{r}}^{\prime }\right)\frac{1}{2}\left({\text{r}}^{\prime }-\text{r}\right)\left({\text{r}}^{\prime }-\text{r}\right):\nabla \nabla C\left(\text{r},t\right). \end{array} \end{array}$$

The next step is to separate the two mechanisms that contribute to the flow of the drug in the brain tissue, namely the diffusion and the pressure-driven convection. We get:

(A4) $$\begin{array}{l} w\left(r,r^{\prime }\right)\;\equiv \;W\left(r^{\prime }-r;r^{\prime }\right)\;\Omega \;\left(p\left(r^{\prime }\right)-p\left(r\right)\right)\\ \;\approx F\left(|r,r^{\prime }|;r^{\prime }\right)\left[\lambda +\frac{1}{2}\left(p\left(r^{\prime }\right)-p\left(r\right)\right)\right]\\ \;=w_{\text{d}}\left(r,r^{\prime }\right)+\frac{1}{2\lambda }w_{\text{d}}\left(r,r^{\prime }\right)\;(p\left(r^{\prime }\right)-p\left(r\right)] \end{array}$$

where *p*(**r**) is the pressure at point **r** and $w_{\text{d}}\left(\text{r},{\text{r}}^{\prime }\right)=\lambda F\left(|\text{r},{\text{r}}^{\prime }|;{\text{r}}^{\prime }\right)$ denotes the contribution to the rates that comes exclusively from the diffusion process. The function Ω contains all of the dependence of the transition rate *w* in the pressure difference. In essence this is an expansion in the pressure difference, where we only keep first order terms. The term *F*(|**r**,**r**′|; **r**′) (*p*(**r**′) − *p*(**r**)) is proportional to the contribution of convection (with a permeability proportional to *F*(|**r**,**r**′|; **r**′)) while the term λ*F*(|**r**,**r**′|; **r**′) is proportional to the contribution of diffusion. The factor λ reflects the difference in the scaling between the convection and the diffusion in brain tissue.

The function *F* encompassing the effects of diffusion can be written as a Taylor series:

(A5) $$\begin{array}{l} F\left(|\text{r},{\text{r}}^{\prime }|;{\text{r}}^{\prime }\right)\approx F\left(|\text{r},{\text{r}}^{\prime }|;\text{r}\right)\left({\text{r}}^{\prime }-\text{r}\right)\cdot \nabla F+\frac{1}{2}\left({\text{r}}^{\prime }-\text{r}\right)\left({\text{r}}^{\prime }-\text{r}\right):\nabla \nabla F. \end{array}$$

A Taylor series expansion for the pressure at point **r**′ gives for the pressure difference:

(A6) $$\begin{array}{l} p\left({\text{r}}^{\prime }\right)-p\left(\text{r}\right)\approx \left({\text{r}}^{\prime }-\text{r}\right)\cdot \nabla p\left(\text{r}\right)+\frac{1}{2}\left({\text{r}}^{\prime }-\text{r}\right)\left({\text{r}}^{\prime }-\text{r}\right):\nabla \nabla p\left(\text{r}\right) \end{array}$$

Combining (A4) with (A5) and substituting the result and (A6) into (A3) gives:

(A7) $$\begin{array}{l} \frac{\partial C\left(r,t\right)}{\partial t}=\nabla \;\cdot \;\left[\theta \frac{R\left(r\right)}{\text{λ}}\nabla p\left(r\right)C\left(r,t\right)+\nabla \;\left(R\left(r\right)C\left(r,t\right)\right)\right] \end{array}$$

where the tensor **R**(**r**) is defined by the equation

(A8) $$\begin{array}{l} \begin{array}{c} \text{R}\left(\text{r}\right)\equiv \frac{1}{2}\underset{{\text{r}}^{\prime }}{\sum }\lambda \;F\left(|\text{r},{\text{r}}^{\prime }|;\text{r}\right)\left({\text{r}}^{\prime }-\text{r}\right)\left({\text{r}}^{\prime }-\text{r}\right)\\ =\frac{1}{2}\underset{{\text{r}}^{\prime }}{\sum }w_{\text{d}}\left(\text{r},{\text{r}}^{\prime }\right)\left({\text{r}}^{\prime }-\text{r}\right)\left({\text{r}}^{\prime }-\text{r}\right). \end{array} \end{array}$$

Equation (A7) is a continuity equation, according to which the time derivative of the concentration of the fluid is equal to the divergence of the total concentration flux, resulting from the diffusive and the convective process. In (31), the permeability for the convection-induced spread of the drug in the anisotropic brain tissue is defined as:

(A9) $$\begin{array}{l} \text{T}\left(\text{r}\right)\equiv \frac{\text{R}\left(\text{r}\right)}{\lambda }\varphi \end{array}$$

where φ is the porosity. Equation (A9) implies that the permeability tensor of the drug in the brain tissue shares the same eigenvectors as its dispersion tensor. Because our knowledge of the comparative scale of the two tensors that appear in Equation (A9) comes from animal data, and we indeed only know the comparative scale of the two, we absorb the porosity φ in the factor λ and set $\hat{\lambda }=\lambda /\phi$, to get

(A10) $$\begin{array}{l} \text{T}\left(\text{r}\right)\equiv \frac{\text{R}\left(\text{r}\right)}{\hat{\lambda }}. \end{array}$$

The term −**T**(**r**)∇*p*(**r**) in (A7) is the velocity field:

$$\begin{array}{l} \text{v}\left(\text{r}\right)=-\text{T}\left(\text{r}\right)\nabla p\left(\text{r}\right), \end{array}$$

so Equation (A7) becomes

(A11) $$\begin{array}{l} \frac{\partial C\left(r,t\right)}{\partial t}=-\nabla \left[v\left(r\right)\;\cdot \;C\left(r,t\right)/\varphi \right]+\nabla \left[R\left(r\right)\nabla \;\cdot \;C\left(r,t\right)\right]. \end{array}$$

Multiplying all terms of Equation (A11) by the porosity and taking into account the fact that the changes in porosity are negligible in this case we finally get

$$\begin{array}{l} \varphi \frac{\partial C(r,t)}{\partial t}=-\nabla \left[v\left(r\right)\;\cdot \;C\left(r,t\right)\right]\;+\;\nabla \left[\varphi \;R\left(r\right)\nabla \;\cdot \;C\left(r,t\right)\right]. \end{array}$$

For an incompressible fluid we additionally have that

$$\begin{array}{l} \nabla \cdot \text{v}\left(\text{r}\right)=0. \end{array}$$

We thus have the system of 3 equations that describe the movement of the fluid:

(A12) $$\begin{array}{l} \nabla \cdot \text{v}\left(\text{r}\right)=0 \end{array}$$

(A13) $$\begin{array}{l} \text{v}\left(\text{r}\right)=-\text{T}\left(\text{r}\right)\nabla p\left(\text{r}\right) \end{array}$$

(A14) $$\begin{array}{l} \varphi \frac{\partial C\left(r,t\right)}{\partial t}=-\nabla \left[v\left(r\right)\;\cdot \;C\left(r,t\right)\right]\;+\;\nabla \left[\varphi \;R\left(r\right)\nabla \;\cdot \;C\left(r,t\right)\right]. \end{array}$$

with Equations (A8) and (A10) needed in order for the formalism to be complete.

We note here that it is possible to follow on and include higher order corrections to the model. Specifically, the Taylor series expansions can be extended to include corrections up to fourth order. In that case, combining the terms as earlier, the third order terms vanish due to symmetries, and we are left with fourth order terms. Even though such terms could, in principle, be useful for some fluid dynamics problems, numerical schemes that deal with fourth-order terms are not widely available, because they tend to be unstable and difficult to converge. It is, therefore, difficult to construct a CFD model that would give reliable predictions that include those higher order terms. Regardless of that fact, for the specific problem of CED in the human brain that we are dealing with, including those higher order terms is not necessary. Including such terms is necessary in tractography algorithms, when trying to distinguish between fiber crossings. However, in our case, because the fluid molecules move in the extracellular space, it is of no relevance whether the encountered anisotropy is a result of crossing, bending or fanning fibers. In other words, we are not interested in extracting the diffusion orientation distribution function, and for that reason we do not include terms of order higher than second in our analysis.
